# Coordinated community structure among trees, fungi and invertebrate groups in Amazonian rainforests

**DOI:** 10.1038/s41598-019-47595-6

**Published:** 2019-08-05

**Authors:** Jason Vleminckx, Heidy Schimann, Thibaud Decaëns, Mélanie Fichaux, Vincent Vedel, Gaëlle Jaouen, Mélanie Roy, Emmanuel Lapied, Julien Engel, Aurélie Dourdain, Pascal Petronelli, Jérôme Orivel, Christopher Baraloto

**Affiliations:** 1 CNRS, UMR Ecologie des Forêts de Guyane, Campus agronomique, BP 316, 97379 Kourou Cedex, France; 2 International Center for Tropical Botany, Department of Biological Sciences, Florida International University 11200 S.W, 8th Street Miami, Florida, FL 33199 USA; 3 INRA, UMR Ecologie des Forêts de Guyane, Campus agronomique, BP 316, 97379 Kourou Cedex, France; 4 Centre d’Ecologie Fonctionnelle et Evolutive (CEFE UMR 5175, CNRS–Université de Montpellier–Université Paul-Valéry Montpellier–EPHE), 1919 Route de Mende, F-34293 Montpellier, France; 5 AgroParisTech, UMR Ecologie des Forêts de Guyane, Campus agronomique, BP 316, 97379 Kourou Cedex, France; 6 Université Paul Sabatier–CNRS-IRD-ENFA, laboratoire Evolution et Diversité Biologique, UMR5174, 118 route de Narbonne, 31062 Toulouse cedex, France; 7 Taxonomia International Foundation, 7 rue Beccaria, 72012 Paris, France; 8 IRD, UMR AMAP, Boulevard de la Lironde, TA A-51/PS2, F-34398 Montpellier Cedex 5, Montpellier, France; 9 CIRAD, UMR Ecologie des Forêts de Guyane, Campus agronomique, BP 316, 97379 Kourou Cedex, France

**Keywords:** Community ecology, Tropical ecology

## Abstract

Little is known regarding how trophic interactions shape community assembly in tropical forests. Here we assess multi-taxonomic community assembly rules using a rare standardized coordinated inventory comprising exhaustive surveys of five highly-diverse taxonomic groups exerting key ecological functions: trees, fungi, earthworms, ants and spiders. We sampled 36 1.9-ha plots from four remote locations in French Guiana including precise soil measurements, and we tested whether species turnover was coordinated among groups across geographic and edaphic gradients. All species group pairs exhibited significant compositional associations that were independent from soil conditions. For some of the pairs, associations were also partly explained by soil properties, especially soil phosphorus availability. Our study provides evidence for coordinated turnover among taxonomic groups beyond simple relationships with environmental factors, thereby refining our understanding regarding the nature of interactions occurring among these ecologically important groups.

## Introduction

The outstanding biodiversity of tropical forests represents a major challenge for ecologists seeking to understand how species are spatially assembled in these ecosystems^1^. Most studies to date have investigated assembly rules within individual species groups, especially plants^2,3^, with less than ten percent of published articles focusing on other living groups between 2008 and 2018 (Google Scholar research using the terms “*community assembly tropical forests*” and “*determinants of community assembly tropical forests*” in separate searches; pages 1–20). At the same time, there has been considerably less investment to examine how the composition of different groups, notably groups of major ecological importance (e.g. primary producers, decomposers, herbivores and their predators), display coordinated turn-over across geographical and environmental gradients. Addressing this topic would shed light on the role exerted by environmental filtering and biotic interactions between communities on biodiversity organisation in the most diverse ecosystem on earth.

The majority of studies examining associations between different taxa have relied on simplified proxies such as diversity indices, often with conservation-oriented purposes. For instance, many works have tested whether tree diversity represents a reliable surrogate for the diversity of other taxa, mainly arthropods and vertebrates^4–7^. Nevertheless, there has only been weak and inconsistent evidence establishing significant diversity associations between taxa in tropical forests^8^, while studies on the subject have often been hampered by difficulties to obtain accurate diversity estimations in these hyper-diverse ecosystems^9^. Moreover, diversity indices provide limited information to unveil the complex assembly rules that structure communities, for which compositional information would be more appropriate^10^. Multi-taxon composition data are considerably lacking in ecology, especially in rainforests, because they require considerable logistical and time-consuming efforts to coordinate the work of specialists of different taxa^11^. Additionally, we are witnessing a sharp decline of trained taxonomists able to identify specimens in many groups. This is particularly problematic in the case of arthropods, which represent most of the macroscopic diversity in the tropics^5^, as well as for other hyper-diverse and poorly known groups including fungi and non-arthropod invertebrates. The few studies reporting compositional association between taxa in tropical ecosystems have involved testing association with tree species^12–14^, which are generally well described and relatively easy to inventory^15^.

Even though empirical evidence is sparse, strong hypotheses can be proposed to link composition between tree communities and other groups in tropical forests. Co-evolution processes have driven trees to offer a huge variation of food supply, anti-herbivore defences and/or housing^16^, and thereby to interact in multiple ways with other communities. In a recent study^10^, strong compositional associations have been emphasized between plant, arthropod and micro-organism communities in a diverse subtropical forest of South China. The study suggested that tree communities exert a bottom-up control on aboveground arthropod communities while, at the same time, trees are influenced by soil micro-organisms and decomposers via a top-down effect. The key role of trees in the functioning of forest ecosystems has also served as an argument to support their usefulness as a surrogate for the composition of other groups, for instance arthropod^17^ and fungal communities^18^.

Further hypotheses can be suggested to link composition among other species groups. Ants for example, are extremely diverse, both ecologically and taxonomically, and play key functional roles in forest ecosystems^19^. They are therefore likely to influence many other communities. Leaf-litter ants, in particular, profoundly impact soil structure through foraging and tunnelling activities, while they also represent major predators of the soil fauna^20^. Spiders also exhibit key functions as predators, and competitive and predator-prey interactions between ants and spiders are likely to be reflected in significant associations between both groups^21^. Other major ecosystem functions, including decomposition of organic matter, nutrient cycling and mycorrhizal associations^22^ are provided by fungi, which represent another highly diverse and understudied group in tropical forests. Fungal communities are expected to interface with tree communities, via feedback interactions between plants and soil^23^ and pathogen-mediated negative density dependent effects^24,25^. Other major decomposers comprise earthworm communities, which are known to be influenced by chemical properties of the litter and soil conditions, but also to substantially modify soil properties and thereby impact other groups^26^.

Taxonomic congruence among species groups may result not only from biotic interactions but also be explained by a similar response to environmental conditions. The assumption that environmental variation (altitudinal, topographical and edaphic) drives beta-diversity in tropical forests has been abundantly demonstrated for trees^2,3^. Such work has built a strong foundation for conservation programs to identify areas of high biodiversity^27^. However, there has been considerably less evidence for the influence of environment among other groups^19,28^. Moreover, very few attempts have been undertaken to examine to what extent coordinated taxonomic turn-overs among different species groups are driven by the filtering of common environmental filters. Previous studies in the Amazon have reported congruence of taxonomic composition between Melastomataceae and Pteridophytes, partly explained by Ca, Mg and K content, and soil texture^29^. More recently, Lemes Landeiro *et al.*^30^ have emphasized the existence of coordinated composition turn-overs among 22 taxonomic groups that were explained by environmental variables, especially soil clay and phosphorus content.

Despite some sparse evidence for cross-taxon congruence in tropical forests, there remains a great need for multi-taxon composition data combined with environmental information to better understand the factors explaining coordinated turn-over of composition among different species groups. This would also help to shed light on the nature of interactions between groups, so as to improve our predictions for the potential consequences of species loss on ecosystem functioning in tropical forests^31^.

The present study aims to (i) investigate congruence of taxonomic composition among five species groups of major ecological importance (trees, ants, spiders, fungi and earthworms), of which some remain critically understudied in the tropics, and (ii) to examine whether coordinated species-turn-over among pairs of groups is explained by soil variables. To do so, we carried out exhaustive and standardized multi-taxa inventories in 36 plots distributed across four remote areas of French Guiana representing the biogeographic and environmental gradients of this hyper-diverse tropical region. We address the following specific questions:

1. (**a**) To what extent is the taxonomic composition of the five living groups correlated to each other? **(b)** How do we interpret the significant pairwise associations?
2. (**a**) To what extent is correlation of composition between groups due to a coordinated response to environmental heterogeneity among plots? **(b)** Is the coordinated response explained by the same environmental variables among pairs of groups?

## Results

### Co-inertia between taxonomic groups

**Figure 1:**
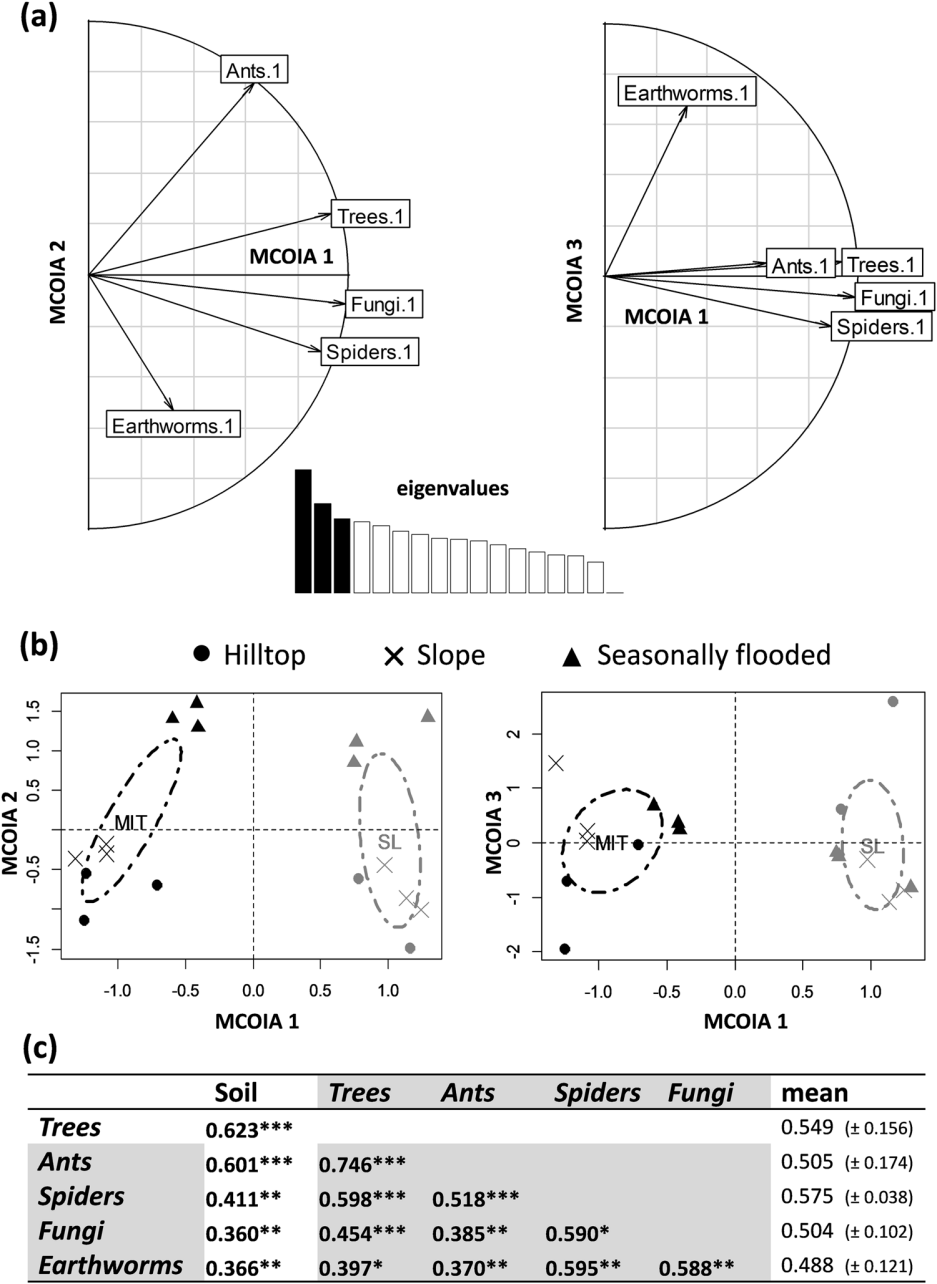
(**a**) Correlations between the first axes of separate PCA (performed on each species group) and the three first axes of the MCOIA (axes 1–2 and 1–3). (**b**) Projection of plot scores on axes 1–2 and axes 1–3 of the MCOIA, emphasizing the two sites where compositional data was available for the five groups (Mitaraka = MIT and Saül-Limonade = SL) as well as topographical habitats (hilltop, slope and seasonally flooded). Histograms represent the eigenvalues of the MCOIA axes. (**c**) Adjusted RV coefficients calculated for each pair of taxonomic groups (portion of the table shaded in grey), and between each taxonomic group and soil variables (first column). Asterisks indicate whether values were significant (in bold) according to the MSR test: *P < 0.05; **P < 0.01; ***P < 0.001. The last column represents the mean (±standard deviation) of RV values per group. The MCOIA performed using other combinations of species groups are presented in Figs S4 and S5.

A Multiple Co-Inertia Analysis (MCOIA), an ordination method allowing visual representation of associations among multiple data tables, was performed to explore patterns of compositional associations among taxa. The analysis showed positive correlation of taxonomic composition between the five species groups (Fig. 1a). A marked difference of the overall composition (all groups together) was observed between the *Mitaraka* and *Saül*-*Limonade* sites on the first MCOIA axis (Fig. 1b). Within each of these sites, differences were also well marked between seasonally flooded soils and the two other habitats (hilltop and slope) on the second axis of the MCOIA (see left graph in Fig. 1b). For simplicity, Fig. 1a,b show the results obtained in the two sites where data for all groups were available, but MCOIA performed with three or two sites (where more than two groups were inventoried) produced redundant results (Figs S4 and S5). Pairwise compositional associations among groups were quantified by the RV coefficient of Co-Inertia Analyses (COIA), which was tested using Moran Spectral Randomisations (MSR) to take spatial autocorrelation into account. RV values were significant (*P* ≤ 0.05) for each pair of groups, regardless of the soil heterogeneity (Fig. 1c). The highest RV value reached 0.746 (*P* < 0.001; MSR test) between trees and ants. Average RV values (calculated over the four values per group) ranged from 0.488 (SD =  ± 0.121) for earthworms to 0.575 for spiders (SD =  ± 0.038). All groups also displayed significant co-inertia with soil variables, with trees and ants showing the highest associations (RV = 0.623 and 0.601, respectively).

RV values were highly correlated to Procrustes residuals sum of squares (PRSS), after removing or not the plots on seasonally flooded forest and by using either taxonomic abundance/occurrence or presence-absence data (with *r*-Pearson values ranging from 0.62 to 0.96; Figs S6 and S7), hence showing that both methods produced consistent results. All RV and PRSS values obtained after removing or not plots on seasonally flooded forest, and using abundance or presence-absence data, are presented in Tables S1 to S4.

### Coordinated response to environmental variables for each pair of groups

Four out of the ten pairs of groups (40%) remained significantly explained (*P* ≤ 0.05) by all soil variables after correcting for multiple tests: trees-ants, trees-spiders, trees-fungi and ants-spiders (Table 1). The relative contribution of each soil variable was quantified using the sum of weighted AICc (corrected Akaike Information Criterion) values (SWA) from a Model Averaging with Multivariate Generalized Linear Model. On average, soil phosphorus availability (P) was the variable that best explained the co-inertia among the four significant pairs (mean SWA = 0.62; SD =  ± 0.03; Table 1). The best following explanatory variable displayed mean SWA values ranging from 0.49 for the percentage of sand to 0.54 and 0.55 for Ca and Mg, respectively. P was the best explanatory variable for each of the four significant pairs (from 0.59 for the trees-fungi to 0.66 for the trees-spiders pair), along with Ca (0.58) and Mg (0.60) for the trees-fungi pair.

**Table 1:** Effect of soil variables on the composition co-inertia for each pair of taxonomic groups.

	S	Trees	Trees	Trees	Trees	Ants	Ants	Ants	Spiders	Spiders	Fungi	
		Ants	Spiders	Fungi	Earthw.	Spiders	Fungi	Earthw.	Fungi	Earthw.	Earthw.	
ASV	BF	**43.78**	**45.54**	**45.24**	25.98	**43.53**	30.47	40.35	16.11	30.82	14.08	**Mean**
Clay	ns	0.50	0.49	0.50	0.51	0.52	0.49	0.50	0.46	0.52	0.49	0.50
Silt	ns	0.51	0.52	0.50	0.51	0.55	0.49	0.52	0.46	0.53	0.51	0.52
Sand	ns	0.50	0.47	0.50	0.51	0.49	0.51	0.47	0.45	0.53	0.47	0.49
OM	**BF**	0.54	0.53	0.49	0.51	0.48	0.50	0.51	0.46	0.58	0.44	0.51
N	**B**	0.53	0.50	0.47	0.47	0.48	0.49	0.52	0.47	0.58	0.44	0.50
AP	**BF**	0.62	0.66	0.59	0.51	0.62	0.57	0.54	0.56	0.53	0.51	0.62
Ca	ns	0.51	0.54	0.58	0.52	0.54	0.54	0.54	0.54	0.53	0.55	0.54
Mg	ns	0.52	0.51	0.60	0.55	0.57	0.53	0.57	0.52	0.60	0.53	0.55
K	**BF**	0.49	0.51	0.49	0.52	0.49	0.46	0.50	0.48	0.52	0.47	0.50
Na	ns	0.51	0.56	0.51	0.48	0.50	0.51	0.46	0.48	0.44	0.43	0.52
Al	ns	0.49	0.49	0.49	0.50	0.52	0.47	0.55	0.47	0.52	0.49	0.50
Mn	ns	0.50	0.56	0.50	0.50	0.49	0.48	0.53	0.47	0.60	0.43	0.51

The first line corresponds to adjusted R² values quantifying the effect of all soil variables (ASV) on the co-inertia. Significant R² values (*P* ≤ 0.05 using a MSR test with a Sidak correction for multiple tests) are indicated in bold. The next lines represent the sum of weighted AIC_c_ (SWA) of each soil variable, calculated from the model averaging for multivariate generalized linear models. S: spatial structure; B = broad-scale (inter-site) spatial structures detected only; BF = both broad- and fine-scale (intra-site) structures detected. ns = no significant structure detected. Mean: mean SWA values (in italics) for the four pairs that were significantly explained by ASV.

P was significantly spatially structured at both fine (within-site) and broad (among-site) spatial scales, as were the overall soil heterogeneity (all soil variables together), OM and K. N only displayed broad-scale spatial structures. No significant structure was detected for soil texture, Ca, Mg, Na, Al and Mn.

## Discussion

We observed significant compositional associations between five species groups that provide major ecological functions in tropical forests. For four out of the ten pairs of groups, the co-inertia between the two groups was significantly explained by the overall soil heterogeneity, with P availability being the most influential variable.

### All pairs of taxonomic groups showed coordinated turn-over of composition

All pairs of taxonomic groups displayed significant co-inertia, reflecting significant coordinated compositional turn-overs across plots, regardless of the influence of soil conditions (Fig. 1c). Two out of the three RV values < 0.5 were obtained with earthworms (the trees-earthworms and ants-earthworms pairs). These relatively low values may have partly resulted from lower statistical power since earthworms’ data were available in two sites only. However, this group was strongly associated with spiders and fungi and thus additional data on earthworms are needed to verify its association with other groups. The relatively low RV value (0.385) observed for the fungi-ants pair may be explained by the absence of ant-associated fungi in our sampling. Indeed, we targeted Basidiomycota and visible fungi, while Chaetothyriales and most ant-associated fungi are leaf-endophytes belonging to Ascomycota. The RV value was nonetheless significant, which may suggest the existence of other interactions between both groups for which we lack hypotheses.

The highest co-inertia (RV = 0.746) was found between trees and ants, while all groups were significantly associated to trees. This result is in agreement with previous studies that emphasized the influence of trees, as key drivers of resource (energy, nutrients) fluxes, on the composition of other communities following bottom-up effects^4,10^. It also supports the idea that tree species composition may serve as a useful surrogate for other communities and thereby contribute to improving current estimations of local and regional tropical diversity which remain greatly inaccurate^32^. The key role of trees in structuring tropical forest ecosystems has often been a foundation for conservation management programs and to help policy makers to choose protected areas and to improve models of biodiversity dynamics in response to climate and land use change scenarios^33^.

The strong association between trees and ants may possibly results from the quality of the litter fall that influences the habitat structure as well as the community of ant preys^34,35^. The significant association obtained between ants and all other groups supports previous evidence for the major ecological functions that they provide in tropical forests^19,20,36,37^. Ant species being well-described in the Amazon region and relatively easy to inventory, our results reveal their utility as another potential proxy in future biodiversity assessment surveys^38^.

RV values were, on average, lower among pairs including trees (mean RV = 0.549) than among pairs including spiders (mean RV = 0.575; Fig. 1c). The latter results may depict a network of interactions that is more complex than proposed by most bottom-up and top-down models^10^, with spiders interacting more directly with the other studied groups than do trees. For example, the significant association observed between spiders and ants (RV = 0.518) may reflect the strong intra-guild competition occurring between the two groups, which both comprise diverse generalist predators^21^. The strong taxonomic congruence between trees and spiders (RV = 0.598) may be explained by the same reasons as we proposed for the tree-ant association (see above). Interactions between spiders and fungi and between spiders and earthworms have never been reported to date and we lack hypotheses to interpret the significant associations observed for these pairs of groups.

The highly significant association between trees and fungi (RV = 0.454) supports previous evidence highlighting the importance and the variety of interactions occurring between these two groups in the Amazon^39^. For example, different fungi guilds (arbuscular mycorrhizal and ectomycorrhizal fungi, as well as saprophytic fungi) have been shown to exert a top-down control on tree species composition in a tropical forest of south-eastern China^9^. Interestingly, changes in fungi communities where also highly correlated with changes in earthworm communities (RV = 0.588). As saprophytic fungi and especially wood-decaying fungi were the most abundant, we can hypothesize that fungi and earthworms share similar niches in tropical forests. Moreover, fungi have already been demonstrated to represent an important food source for earthworms, and different earthworm species can have preferences for distinct fungal taxa^40^.

It is worth pointing out that compositional associations were observed despite uneven taxonomic resolution among groups (species for ants and trees, genera for fungi and spiders, OTU’s for earthworms). This makes sense when considering previous evidence reporting that genera represent good ecological surrogates for both spiders^41^ and fungi^42^. In the case of earthworms, current species delimitations have been suggested to be more precise when considering the molecular classifications we employed to determine OTUs, due to substantial cryptic morphological variation among putatively distinct taxa^43^.

### Coordinated responses to edaphic heterogeneity among pairs of groups

Four out of the ten pairs of groups significantly reacted in a coordinated way with the overall edaphic variation (Table 1). The absence of signal for the four pairs of groups that included earthworms might result from low statistical power due to the fewer number of sites (two) in which they were inventoried. It may also be due to a limited effect of the soil stoichiometry variation on these decomposers compared to the role exerted by the carbon quality of the soil^44^.

P was the soil factor that best explain the coordinated response among the four pairs significantly explained by the overall soil heterogeneity (mean SWA = 0.62). The significant effect of P on coordinated taxonomic turn-overs has rarely been emphasized before, but a previous study^30^ demonstrated that the availability of this element, along with the percentage of clay, explained the congruence between lianas of the Bignoniaceae family and other living groups in the Brazilian Amazon. We may assume that the joint influence of P and other soil properties result from bottom-up effects exerted by trees on the composition of other groups: it has been well supported that tree species composition varies with soil P availability^45^ and other soil variables^3^, thereby influencing other groups depending at least partially on tree communities for resource use. Soil texture was not among the variables that best explained compositional congruence among pairs of groups, although the influence of this property on tree community composition^46^ but also on ant community composition^34^ has been well established in tropical forests. This may be due to the low soil textural variation among plots (the coefficient of variation ranged from 0.46 to 0.74 for the three soil texture variables, while it reaches, on average, 1.18 among all the other variables). In a second position, we found that compositional congruence among groups was also well explained by Mg and Ca (mean SWA over the four pairs = 0.55), for which the role has, to our knowledge, never been demonstrated for explaining coordinated beta-diversity patterns in tropical forests. Nevertheless, there has been previous evidence for its influence on composition within individual groups, especially in rainforest tree^2^ and fungi^28^ communities.

### Questions raised and research perspectives

In light of our results and the previous works that have highlighted compositional associations among other groups than the ones studied here^10–14^, it is cogent to consider to what extent taxonomic congruence occurs among other communities in the ecosystem, such as vertebrates, herbaceous and epiphytic plants, and other micro-organisms. For instance, we could hypothesise that birds and mammals display coordinated beta-diversity with most of the taxa investigated in our study, e.g. via predation on invertebrates and plant use (herbivory, zoogamy, zoochory). However, the disparities of dispersal capacities between large and small animals would jeopardize the chance of detecting compositional associations at the 1.9 ha scale of our plots^47^. Sampling designs should therefore take multiple spatial scales into account so that congruent patterns between animal groups displaying contrasted spatial structures are analysed at appropriate spatial scales. One means to accomplish this will be to integrate the recent progress in assessing biodiversity using metabarcoding methods^48^, which represent a promising approach for investigating compositional association between macro and micro-organism communities. We hope that our work will encourage further similar studies in tropical forests but also extend our approach to other ecosystems, in order to better understand the importance of coordinated beta-diversity and the underlying mechanisms driving these patterns in natural communities.

## Conclusion

We demonstrate the existence of coordinated beta-diversity patterns among five species groups of major ecological importance in tropical forests, a rarely investigated subject in ecology. The coordinated species turn-overs partly and significantly resulted from the common effect of soil variables, especially soil phosphorus availability, but were also partly unrelated to the heterogeneity of the measured soil variables. Our results shed new light in our understanding of species assembly rules among groups of which some remain critically understudied in the tropics. They also confirm previous studies reporting the usefulness of trees but also ants as proxies for assessing the composition of other species groups, which will improve the design and impact of conservation programs using tree and arthropod inventories in tropical forests. Our study paves the way for deeper investigations of biotic interactions in the tropics and in other ecosystems, and may bring new hypotheses to understand biodiversity patterns and even diversification processes.

## Materials and Methods

### Study area and inventory sites

Between 2012 and 2017, multi-taxonomic inventories were carried out in four sites of mature lowland and lower mountain tropical moist forests across French Guiana (Fig. S1). French Guiana is located in the northern part of South America, between latitudes 2° and 6°N, and between longitude 51° and 55°W. Soils of the region are heavily weathered and highly depleted in soil nutrients. The relief is extremely eroded and generally flat, with elevation rarely exceeding 200 m, except in two mountain ranges with peaks beyond 800 m. Mean annual rainfall in the four inventory sites ranges between 1500 and 3000 mm and is distributed seasonally throughout the year^49^. The wet season stretches from December to July, and is usually interrupted in February or March by a short dry period, while the dry season occurs from August to November with monthly rainfall rarely exceeding 100 mm. Mean temperature oscillates around 25 °C with low seasonal variation^49^.

The four inventory sites comprised six to 12 plots of 1.9 ha each (see next section for details), spaced by at least 500 m to each other, and located on habitats presenting contrasted topographical features^50^: hilltop, slope and seasonally flooded forest (Table 2 and Appendix S1 for details). The most northerly site, *Trinité* (4°24′36″N, 53°24′47″W), comprised six plots, four on hilltops and two on seasonally flooded forest. In the second site, *Saül-Limonade* (3°33′36″N, 53°12′W), 12 plots were inventoried, four on each topographical habitat. In the third site, *Itoupé* (3°1′12″N, 53°6′W), nine plots were disposed along an altitudinal gradient, at 400, 600 and 800 m (three plots per elevation level). In the fourth and most southerly site, *Mitaraka* (2°13′12″N, 54°27′36″W), nine plots were inventoried, three on each topographical habitat. *Saül-Limonade*, *Itoupé*, *Mitaraka* are part of the National Amazonian Park of French Guiana (PAG, www.pag.fr). *Trinité* is a natural reserve part of the Network of Natural Reserve of French Guiana (www.guyane-parcregional.fr).

**Table 2:** Characteristics and taxonomic diversity of each living group in all study sites combined and within each site. *nd*: no data available.

	All sites	Itoupé	Mitaraka	Saül-Limonade	Trinité
*Total Nr of plots*	36	9	9	12	6
*Plots on plateau*	15	4	3	4	4
*Plots on slope*	12	5	3	4	0
*Seasonally flooded plots*	9	0	3	4	2
*Altitudinal range (m)*	110–800	300–800	201–280	306–445	110–430
**Taxonomic richness**					
*Tree species*	1054	**436**	364	322	400
*Fungi genera*	171	81	72	**92**	61
*Ant species*	445	**276**	273	186	152
*Spider genera*	81	50	**58**	56	*nd*
*Earthworm OTU’s*	65	*nd*	27	**44**	*nd*
***ENT***_**2**_					
*Tree species*	127.4	**118**.**7**	54.6	46.2	176.4
*Fungi genera*	22.7	19.3	**21**.**9**	16.2	10.4
*Ant species*	77.5	**65**.**3**	64.3	54.3	46.5
*Spider genera*	11.3	8.1	**13**.**5**	9.5	*nd*
*Earthworm OTU’s*	14.4	*nd*	**9**.**1**	7.6	*nd*

*ENT*_2_ = Effective Number of Taxa expected for a random sampling (with replacement) of 2 individuals, following Dauby & Hardy (2011). Bold values represent the highest taxonomic richness and *ENT*_*2*_ among sites for each living group.

### Plot configuration and taxonomic inventories

Plots were designed using a modification of Gentry plots^51,52^. The protocol consists of establishing ten parallel transects of 10 × 50 m emanating perpendicular to a 200 m central line (every 20 m along this line), successively oriented in alternate directions. In this sampling design, five taxonomic groups were inventoried over different surfaces: tree species (1.9 ha), leaf-litter ants (hereafter “ants” for simplicity, 0.12 ha), spiders (0.12 ha), fungi (1 ha) and earthworms (1 ha). Voucher and tissue samples were collected for all taxa sampled and used to inform the taxonomic hypotheses for species lists in each site. These samples are stored in local laboratories in Kourou and are available for consultation upon request from the authors, with duplicates deposited in regional taxonomic museums (and herbaria for trees). The sampling intensity differed across groups (see details in Appendix S1), with composition data available in the four sites for soil variables, trees, ants and fungi, three sites for spiders and two sites for earthworms. The protocol used to inventory each taxonomic group in the field and identify taxa is detailed in Appendix S1. We identified among all sites: 1054 tree species, 171 fungi genera, 445 ant species, 81 spider genera and 65 OTU’s of earthworms. Table 2 summarizes the overall taxonomic diversity among sites and within each site, represented by the species richness and the effective number of species expected from a random sampling of 2 individuals. The latter statistic was used to provide a diversity measure that gives relatively more weight than species richness to the most abundant taxa^53^.

### Soil and topographical data

Ten bulked soil cores were collected at 0–10, 10–20 and 20–30 cm depth in each plot, at each of the ten crossing points between parallel transects and the main central line (Appendix S1). They were combined into a composite 500 g sample. The latter was dried at 25 °C to reach a constant mass, then sieved to 2 mm and shipped within 3 months for physical and chemical analyses at CIRAD (France). Physical and chemical soil properties were then measured using standard soil analysis protocols^54^. Physical properties corresponded to soil texture (percentage of clay, silt and sand). Chemical variables corresponded to the percentage of soil organic matter (OM), the available content of phosphorus, N and six bioavailable cations (Ca, Mg, K, Na, Al and Mn). After eliminating outlier values^55^, the soil heterogeneity among plots was decomposed using a Principal Component Analysis (PCA) on the correlation matrix between normalised (Box-Cox transformation) centred-reduced soil variables values (Figs S2 and S3). The PCA showed that most plots located on seasonally flooded habitat displayed soil conditions that were very different from the two other habitats (Fig. S2). These habitats differences were therefore emphasized in the ordination analyses described in the following section.

### Data analysis

#### Testing Associations between data tables

In order to quantify and test the compositional associations among pairs of taxa (question 1a), we first used a *Multiple Co-Inertia Analysis* (MCOIA)^56^ to have a visual overview of the correlations between taxonomic groups, and to detect overall compositional (all groups confounded) structures in our data. Abundance and occurrence data were Hellinger-transformed^57^ prior to all analyses to reduce the weight of overly abundant taxa in each group.

We then performed “classical” *Co-Inertia Analysis* (COIA)^58^ to quantify pairwise associations of composition between data tables. The *RV coefficient*^59^ was calculated (at the plot level) to quantify the co-inertia of taxonomic composition between groups and the co-inertia between the composition of each group and soil variables. RV values were adjusted^60^ to avoid biases due to different numbers of columns between the two compared composition matrices. The RV coefficient varies between 0 and 1, a value of 1 indicating that compared matrices are perfectly correlated.

To test RV values while taking spatial autocorrelation into account, we compared the observed coefficients with 999 null values obtained using *Moran Spectral Randomizations* (MSR)^61^. MSR is a flexible method providing a way to generate artificial variables displaying spatial structures that accurately mimic the original structures. To do so, the method first consists in fitting *Moran Eigenvector’s Maps* (MEM)^62^ to univariate or multivariate data, in order to detect spatial patterns at multiple spatial scales. MEM correspond to eigenvectors modelling multi-scale spatial structures in any univariate or multivariate data^62^. Then, based on which plots are connected in the “best” spatial weighting matrix (i.e., the matrix that produce the set of MEM that best fit the data^63^), the MSR method uses a conditional simulation procedure that preserves the original structure of the data^61^. The spatial connections between plots in the spatial weighting matrix were modelled using graph-based configurations (*Gabriel’s* graph and *minimum spanning tree*) which have been demonstrated to be well-suited for modelling spatial structures with nested sampling designs like ours (e.g. distant sites containing six to 12 plots)^63^. A more detailed description of the method used to obtain MEM and perform the MSR test of RV values is described in Appendix S2.

RV coefficients were considered significant when no more than 5% of the null RV values were equal to or higher than the observed value. A significant RV between two groups indicates a significant coordinated turn-over of composition. We took multiple test effects into account in all of our tests by using a Šidák correction^64^.

It is worth noting that conceptually, COIA is very close to the *Procrustes Analysis* which usually leads to redundant results^65,66^. We thus performed Procrustes analyses (calculation of the Procrustes residual sum of square to quantify association between data tables^67^) as a complementary approach to further verify consistency in our results.

#### Testing coordinated response to soil variables for each pair of groups

In order to test the influence of environmental heterogeneity in explaining coordinated species turn-overs among pairs of taxa (questions 2a and 2b), we used the soil data and the co-inertia axes obtained from a COIA performed on each pair of taxonomic groups. We then computed the R² value (adjusted to account for the number of explanatory variables) of a Redundancy Analysis (RDA) of the co-inertia axes on the set of all soil variables. This value was tested by comparing it with 999 null values obtained using the MSR method described above to account for spatial autocorrelation in the residuals of the RDA model. Tests were considered significant if less than five percent of the null values were higher than the observed one. The relative effect of each individual variable was quantified using the sum of weighted AIC_c_ (corrected Akaike Information Criterion) values (hereafter, SWA) from a *Model Averaging with Multivariate Generalized Linear Model*^68^. The R² test and SWA values allowed testing whether the co-inertia between two groups was explained by soil heterogeneity, and if so, which soil variable(s) best explained it. Finally, MEM were also used to test at which spatial scale(s) the overall soil heterogeneity and each individual soil variable explained the co-inertia among pairs of groups (see Appendix S2 for details). Indeed, MEM eigenvalues are directly correlated to Moran’s *I* and therefore to the spatial scales at which variables are structured.

All analyses were performed in the R statistical environment^69^, using packages *car*, *ade4*, *adespatial*, *usdm*, *cocorresp*, *MatrixCorrelation*, *vegan*, *ape* and *mglmn* (see references of these packages in Appendix S2). The R script as well as the soil and composition data tables used to perform our analyses are provided in Appendices S3 to S9.

## Supplementary information

Appendices S1 and S2, supplementary figures S1 to S7 and Tables S1 to S4.

data tables

R code

## Data Availability

The authors confirm that, should the manuscript be accepted, the data supporting the results will be archived in an appropriate public repository such as Dryad or Figshare and the data DOI will be included at the end of the article.
