# Supplementary material for: Coordinated community structure among trees, fungi and invertebrate groups in Amazonian rainforests
Source: Sci Rep. 2019 Aug 5;9:11337. doi: 10.1038/s41598-019-47595-6 (PMC6683196; doi:10.1038/s41598-019-47595-6)
Supplement: Supplementary file 1 — Appendices S1 and S2, supplementary figures S1 to S7 and Tables S1 to S4. [file 41598_2019_47595_MOESM1_ESM.pdf]

Supporting information for the article "*Coordinated community structure among five taxonomic groups in Amazonian rainforests*" – Scientific reports.

Author list

Jason Vleminckx*[1,2], Heidy Schimann[3], Thibaud Decaëns[4], Mélanie Fichaux[1], Vincent Vedel[3], Gaëlle Jaouen[5], Mélanie Roy[1], Emmanuel Lapied[6], Julien Engel[2,7], Aurélie Dourdain[8], Pascal Petronelli[8], Jérôme Orivel[1], Christopher Baraloto[1,3].

*Correspondence to jasvlx86@gmail.com

Author affiliations

1. CNRS, UMR Ecologie des Forêts de Guyane, Université de Guyane, Université des Antilles, Campus agronomique, BP 316, 97379 Kourou Cedex, France.

2. International Center for Tropical Botany, Department of Biological Sciences, Florida International University 11200 S.W. 8th Street Miami, FL. 33199, USA.

3. INRA, UMR Ecologie des Forêts de Guyane, Université de Guyane, Université des Antilles, Campus agronomique, BP 316, 97379 Kourou Cedex, France.

4. Centre d'Ecologie Fonctionnelle et Evolutive (CEFE UMR 5175, CNRS–Université de Montpellier–Université Paul-Valéry Montpellier–EPHE), 1919 Route de Mende, F-34293 Montpellier, France.

5. AgroParisTech, UMR Ecologie des Forêts de Guyane, Université de Guyane, Université des Antilles, Campus agronomique, BP 316, 97379 Kourou Cedex, France.

6. Taxonomia International Foundation, 7 rue Beccaria, 72012 Paris.

7. AMAP, IRD, CIRAD, CNRS, Université de Montpellier, INRA, Boulevard de la Lironde, TA A-51/PS2, F-34398 Montpellier Cedex 5, France.

8. CIRAD, UMR Ecologie des Forêts de Guyane, Université de Guyane, Université des Antilles, Campus agronomique, BP 316, 97379 Kourou Cedex, France.

Appendix S1. Further details on the study area and protocols used to inventory each taxonomic group on the field and identify taxa.

Study area

Between 2012 and 2017, multi-taxonomic inventories were carried out in four sites of mature lowland and lower mountain tropical moist forests across French Guiana (**Fig. S1**). French Guiana is located in the northern part of South America, between latitudes 2 and 6°N, and between longitude 51 and 55°W, at the eastern edge of the Guiana shield. The latter corresponds to an old (ca. 1.8 billion-year) Precambrian tableland covering, almost entirely, the territories of French Guiana, Surinam and Guyana, and extending to the half eastern part of Venezuela, a small portion of eastern Colombia, and the extreme north of Brazil. Soils from the region are heavily weathered and highly depleted in soil nutrients. The relief of French Guiana is extremely eroded and generally flat, with elevation rarely exceeding 200 m, except in some few mountain ranges with peaks beyond 800 m. Mean annual rainfall ranges between 1500 and 3000 mm and is distributed seasonally throughout the year (Gourlet-Fleury 2004). The wet season stretches from December to July, and is usually interrupted in February or March by a short dry period, while the dry season occurs from August to November with monthly rainfall never exceeding 100 mm. Mean temperature oscillates around 25°C with low seasonal variation (Gourlet-Fleury 2004).

Our four inventory sites were composed of six to 12 plots of 2 ha each (see next section), spaced by at least 500 m to each other, and located on habitats presenting contrasted topographical features, following Ferry (2010) – hilltop, slope and seasonally flooded (see Table 2). Hilltop correspond to a habitat situated on the upper part of a hill with vertical water drainage; slope habitat consists in a slope where superficial lateral drainage occurs; a

seasonally flooded habitat is situated in a bottomland that is regularly flooded during rainy season with a water table always observed above 60cm depth and present at the soil surface during at least two consecutive months.

In the first inventory site, *Limonade* (3°33'36"N, 53°12'W), 12 plots were inventoried, four on each topographical habitat. Plots disposed on slope or hilltops were established, when possible, on white sand and terra firme to maximize soil texture contrasts. In the second site, *Itoupé* (3°1'12"N, 53°6'W), nine plots were disposed along an altitudinal gradient ranging from 400, 600 and 800 m (three plots per elevation level). In the third site, *Mitaraka* (2°13'12"N, 54°27'36"W), nine plots were inventoried, three on each topographical habitat. The fourth site *Trinité* (4°24'36"N, 53°24'47"W), consisted of six plots, four of them being located on hilltops and the two others on seasonally flooded habitat. *Limonade*, *Itoupé*, *Mitaraka* are part of the National Amazonian Park of French Guiana (PAG, www.pag.fr). *Trinité* is a natural reserve part of the Network of Natural Reserve of French Guiana (www.guyane-parcregional.fr).

Plot configuration and taxonomic inventories

Plots were designed using a modification of the Gentry plot proposed by Phillips et al. (2003) and described in Baraloto et al. (2013; see also Fig. 2 in Rockwell et al. 2014). The protocol consists of ten parallel transects of 2 × 50 m starting perpendicularly from a central line (every 20 m along this line), successively oriented in alternate directions (see **Figure SA1** below). This sampling delimits an area of 1.9 ha (delimited by a dotted green line on Fig. SA1, within which five taxonomic groups were inventoried: tree species, leaf-litter ants (hereafter “ants” for simplicity), spiders, fungi and earthworms. Composition data (at the plot level) was available in the four sites for soil variables, trees, ants and fungi, three sites for spiders

and two sites for earthworms. These data corresponded to species abundance for trees, species occurrence for ants, genus occurrence for spiders and fungi, and OTUs occurrence for earthworms.

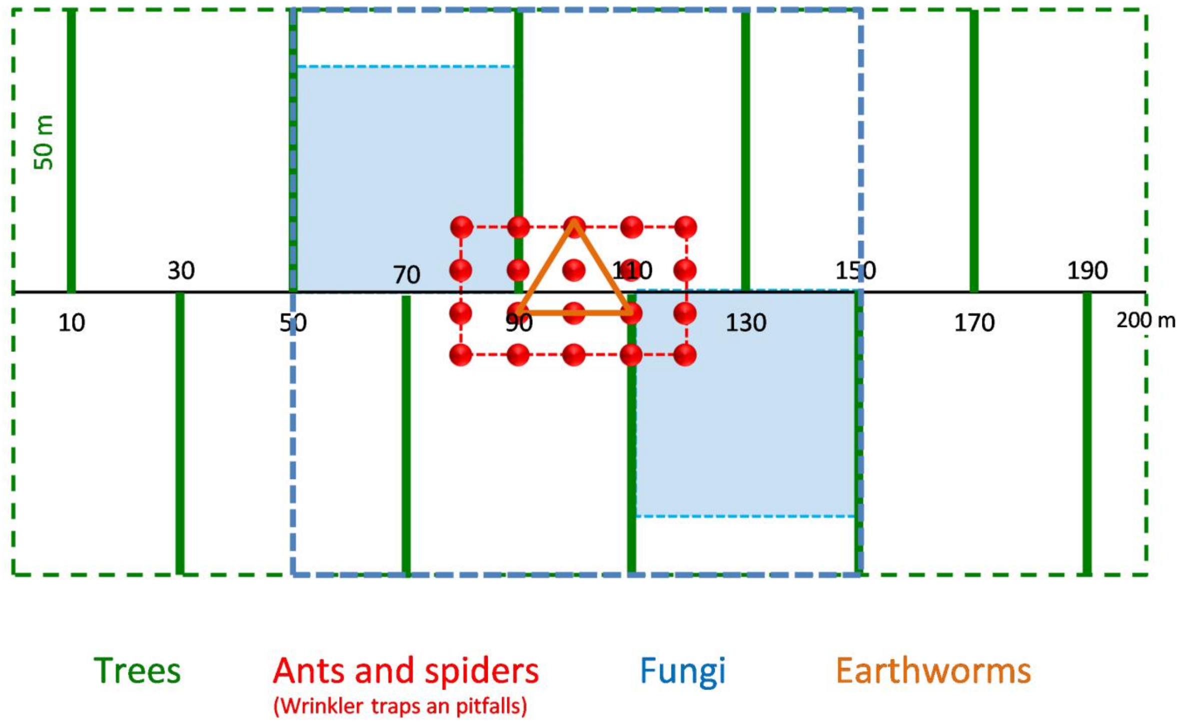

Fig. SA1. Configuration of the modified Gentry plot used to inventory each of the five taxonomic groups.

Trees

All stems with diameter at breast Height (dbh) ≥ 2.5 cm were inventoried along the ten 1 × 50 m-long parallel transects (continuous green lines in **Figure SA1** above). Field identification was done by Engel and Baraloto. Voucher specimen were collected at least once for each putative distinct species per plot in the field, and collected for all individuals that were not identified with complete agreement by the botanists in the field. Duplicate vouchers are

currently stored in reference collections of UMR EcoFoG (Kourou), IRD (Montpellier), and/or ICTB (Miami).

Inventories resulted in a dataset of 1083 distinct taxa in the 36 plots distributed in the four inventory sites, with 322 to 436 distinct taxa observed per site (Table 2). The most abundant species were *Euterpe oleracea* (family: Arecaceae; 11.9% of all stems), followed by *Tetragastris altissima* (Burseraceae; 2.1%) and *Guarea pubescens* (Meliaceae; 1.8%). The most abundant family was the Arecaceae (due primarily to the abundance of *Euterpe oleracea*; 14.4% of all stems), followed by the Fabaceae (11.8%) and the Burseraceae (7.1%). Tree species abundance data are available in Appendix S4.

Ants

Inventories were carried out within an area of 30 x 40 m inside of the Gentry plot (red area in Fig. SA1), within which 20 sampling points were established following a grid-like structure (with 10 m between adjacent points) (**Fig. SA1**), according to protocol described in Agosti and Alonso (2000). Ants were collected at each sampling point using pitfall traps and the Winkler protocol (see Bestelmeyer et al. 2000). Pitfall traps correspond to cylindrical containers of 6 cm diameter each, disposed in the ground and open at the soil surface. Containers were partially filled with a solution composed of water, soap and salt. They were left open for 72 hours. Leaf litter samples were also collected at each sampling point, where ants were collected using mini-Winkler extractors. In the latter, the litter is placed in a mesh bag that hangs inside a cotton enclosure. A -recipient containing ethanol (70%) is placed at the bottom of the cotton enclosure to capture insects dropping from the mesh bag, and so during a period of 48 hours (Bestelmeyer et al. 2000). Combining pitfall traps and Winkler

extractors have been shown to be a reliable method for sampling most of the local diversity of leaf-litter ant species (Delabie et al. 2000; Silva et al. 2013).

Specimen identification

All collected ant specimens were identified at the species or morphospecies level whenever possible. We deposited voucher specimens in the Laboratorio de Mirmecologia, Cocoa Research Centre CEPEC/CEPLAC (Itabuna, Brazil), under the references #5761 and #5762 for mini-Winkler and pitfall traps, respectively. For ant genera with recurrent morphological identification issues (e.g. Pheidole and Solenopsis), we sequenced the 16S rRNA gene for up to four specimens per morphospecies (see Kocher et al. 2016 for details). This protocol allowed us to discriminate specimens that were grouped within the same morphospecies or to gather different morphospecies into a single one.

These inventories resulted in a dataset of 455 identified species, belonging to 65 genera. The most abundant species (many species were assigned a morphotype name) belonged to the genera *Pheidole* (Myrmicinae), *Strumigenys* (Myrmicinae) and *Nylanderia* (Formicinae). Ants species occurrence data are available in Appendix S5.

Spiders

The sampling of spider specimens was carried out at day and night time, using four different methods described hereafter to collect spiders within three vertical layers: the leaf litter (LL), the low underwood vegetation (LUV; 0.1 to 1.5 m above the litter) and the high underwood vegetation (HUV; 1.5 to 2.5 m).

Method 1: reaping (stratum sampled: LUV)

Using a reaping net (diameter: 0.5 m), one collector reaped the LUV during 15 minutes along each of the four lines formed by the five points line up the long axis of the red area in **Fig. SA1**. This sampling was performed once at day time and once at night time, thus resulting to a total of eight samplings (total sampling effort of two hours). This technique allows sampling numerous spiders in activity or not, hidden in the LUV.

Method 2: threshing (stratum sampled: HUV)

This method consists of using a stick and a 1 × 1 m threshing cloth. It is applied along the same lines and during the same time period as method 1. This technique allows sampling numerous spiders in activity or not, hidden in the HUV.

Method 3: hunting the ground at sight (stratum sampled: LL)

During a one-hour period at night time, one collector randomly wanders over the entire plot surface (the green area in **Fig. SA1**) and collect all visible spiders on the LL, using a headlamp and pliers of different sizes to catch them. There is thus one sampling per plot using this method. This technique is particularly efficient to capture nocturnal hunting spiders (Families: Araneidae, Ctenidae, Corinnidae, Theraphosidae, Gnaphosidae etc..) that leave their burrow at night.

Method 4: hunting on tree trunks at sight (strata sampled: LUV and HUV)

This method follows the same protocol as for method 3, except that the collector only captures specimens on tree trunks. The latter represents a particular micro-habitat hosting a species composition that is different from the one found on the ground (main families: Pisauridae, Selenopidae, Sparassidae).

These four methods resulted in a datasets of 81 genera in three of the four inventory sites. The Field sampling and the identification of collected specimens were done by Dr Vincent Vedel. The most abundant genus in all sites was *Ctenus* (family: Ctenidae; 10.6% of the genus

occurrence among plots), followed by *Tmarus* (Thomisidae; 8.4%) and *Cupiennius* (Ctenidae; 7.0%). The genus occurrence data sheet is available in Appendix S6.

Fungi

Fungi fruiting bodies were collected using an easily and reproducible field procedure, carried out in a 1-ha squared subplot located within the 1.9-ha area of the modified Gentry plot (see blue area in **Figure SA1** above). Inventories were conducted as followed: sporocarps were collected by two collectors within three 20 × 20 m quadrats randomly distributed within the 1-ha area. The sampling was standardised to last a maximum of 1.5 h per quadrat. We did not inventory hypogeous fungi which were rarely observed but instead focused on Basidiomycota. A picture of all visible sporocarps was taken. All collected samples were air-dried and numbered in situ. Taxonomic identification on the field was undertaken by Mélanie Roy (CNRS, Toulouse, France) et Gérald Grühn (ONF, France) while further verification and identification (at the genus level) was conducted from vouchered specimens by Anne-Mieke Verbeken (Ghent University, Belgium), Felipe Wartchow (Universidade Federal da Paraíba, Brazil) and Bart Buick (MNHN-CNRS, France). The nomenclature and homogeneity of all taxonomic names were then verified. All collected voucher specimens were stored in the following herbaria: LIP, University of Lille; PC, Paris Natural History Museum; MUCL, Catholic University of Louvain. These inventories allowed collecting a total of 171 genera in the 36 plots distributed across the four inventory sites, with 58 to 74 genera observed per site (Table 2). The most abundant genus was *Marasmius* (family: Marasmiaceae; phylum: Basidiomycota; 14.8% of all identified sporocarps), followed by *Mycena* (Tricholomataceae; Basidiomycota; 6.6%) and *Trametes* (Polyporaceae; Basidiomycota; 6.2%). The genus occurrence data sheet is available in Appendix S7.

Earthworms

<u>Sampling protocol</u>

Within each plot a surface of 1 ha was delimited around on a geo-located point. The following sequence of sampling is carried out:

(1) Three blocs of soil of 25 x 25 cm surface and 20 cm depth, located at the extremities of a 20 m-side triangle (orange triangle in Fig. SA1), are dug out and hand sorted on a plastic cover. All earthworms (including adults, juveniles and cocoons) are collected and kept in three distinct jars, which are referred as the three “quantitative samples”.

(2) An approx. 1 m^2 surface is rapidly hand sorted at any place within the triangle, trying to find a place where large casts are visible on the soil surface (when available). Depth for this sample is minimum 20 cm, but it is interesting to try to access deeper soil layer (up to 50cm) when possible in order to try to collect deep-burrowing species that are never found close to the surface. All earthworms found in this sample are fixed in another jar and referred to the “qualitative soil sample”.

(3) Earthworms are then sought in all available and attainable micro-habitats during a fixed period of at least 3 researcher-hours within the 1ha area (e.g. 1:30h by two people). All life stages (i.e. adults, juveniles and cocoons) are collected, and all types of micro-habitats considered suitable for earthworms were prospected: (3.1) sandy to muddy sediments of stream banks are dug out and hand-sorted; (3.2) litter accumulations and decaying trunks on the soil surface are prospected by carefully sorting them with a small spade or a machete; (3.3) ‘epiphytic soils’ (i.e. organic matter accumulation in epiphytic plants and hollow trees), when attainable, are brought to ground level to be hand-sorted. Specimens collected are

fixed in separate jars corresponding to the microhabitat in which they were found (i.e. river bank sediments, decaying trunks, epiphytic soils, termite nests, etc) and labeled accordingly. It is important during qualitative sampling to consider all earthworm types (e.g. some species of minute size – about 1cm – are easily lost) and life stages (from adults to juveniles and cocoons).

Earthworm conservation and labelling:

Specimens are fixed in pure ethanol, or at least ethanol with >95% concentration, and kept in a freezer prior to tissue sampling. Take care at not putting too much earthworms in a single jar (not more than half of the jar filled with earthworms) and change the ethanol at least once 24h after the first fixation to insure a good preservation. If necessary, divide a given sample into several jars to allow this minimal amount of ethanol. Earthworms fixed correctly must be stiff.

Once to the lab:

Once to the lab, specimens are sorted conservatively into morphospecies (i.e. groups of individuals with similar size, pigmentation and general morphology). Adults and juveniles are systematically considered as different morphospecies because at this stage we lack any reliable character to group them together. Then up to 4 specimens per morphospecies are separated for DNA barcoding, labelled with an individual Id number (the one that will link the barcode to the voucher once sequencing will be performed) and kept in an individual jar, tube, or eppendorf, depending on its size, with ethanol. Tissue sampling has to be done according to the protocol of the iBOL project. Earthworms inventories resulted in a datasets of 71 OTUs in two sites, *Saül-Limonade* (12 plots) and *Mitaraka* (nine plots) (Earthworms OTUs occurrence data).

References for Appendix S1

Agosti D. & Alonso L. (2000). The ALL Protocol. In: Agosti D, Majer J, Alonso L, Schultz T (eds) *Ants: Standard Methods for Measuring and Monitoring Biodiversity*. Washington D.C. USA, pp 204–206.

Baraloto, C., Molto, Q., Rabaud,S.B., Hérault, R. Valencia, L. Blanc, P. V. A. Fine, and J. Thompson. (2013). Rapid simultaneous estimation of aboveground biomass and tree diversity across Neotropical forests: A comparison of field inventory methods. *Biotropica*, 45, 288–298.

Bestelmeyer, B.T., Agosti, D., Alonso, L.E., *et al*. (2000). Field techniques for the study of ground-dwelling ants: an overview, description, and evaluation. In: Agosti D, Majer J, Alonso L, Schultz T (eds) Ants: Standard methods for measuring and monitoring biodiversity. Washington D.C. USA, pp 122–144.

Delabie, J.H.C., Fisher, B.L., Majer, J.D. & Wright, I.W. (2000). Sampling effort and choice of method. In: Agosti D, Majer J, Alonso L, Schultz T (eds) Ants - Standard methods for measuring and monitoring biodiversity. Washington D.C. USA, pp 145–154.

Ferry, B., Morneau, F.O., Bontemps, J.-D., Blanc, L. & Freycon, V. (2010). Higher treefall rates on slopes and waterlogged soils result in lower stand biomass and productivity in a tropical rain forest. *J. Ecol*., 98, 106–116.

Gourlet-Fleury, S., Guehl, J.M. & Laroussinie, O. (2004). *Ecology and management of a neotropical rainforest: lessons drawn from Paracou, a long-term experimental research site in French Guiana* (eds S Gourlet-Fleury, J-M Guehl, and O Laroussinie). 326p.

Kocher, A., Gantier, J.-C., Gaborit, P., *et al*. (2016). Vector soup: high-throughput identification of Neotropical phlebotomine sand flies using metabarcoding. *Mol. Ecol. Res*. 17, 172–182. doi: 10.1111/1755-0998.12556

Phillips, O.L., Vasquez Martinez, R., Nunez Vargas, P., Lorenzo Monteagudo, A., Chuspe Zans, M.E., Galiana Sanchez, W. *et al*. (2003). Efficient plot-based floristic assessment of tropical forests. *J. Trop. Ecol*. 19, 629–645.

Rockwell, C.A., Kainer, K.A., Neves d'Oliveira, M.V., Staudhammer, C.L. & Baraloto, C. (2014). Logging in bamboo-dominated forests in southwestern Amazonia: Caveats and opportunities for smallholder forest management. *Forest Ecol. Manag*., 315, 202–210.

Silva F.H.O., Delabie J.H.C., dos Santos, G.B. *et al* (2013). Mini-Winkler extractor and pitfall trap as complementary methods to sample Formicidae. *Neotrop. Entomol*., 42, 351–358. doi: 10.1007/s13744-013-0131-7.

Appendix S2. Further details on the data analyses section in the Material and Methods: (1) description of co-inertia analyses and the method used to generate *Moran's Eigenvector Maps* and *Moran Spectral Randomisation* tests of the RV values. (2) Description of the method to test coordinated response to soil variables for each pair of groups.

Data analysis

Testing Associations between data tables

In order to quantify and test the compositional associations among pairs of taxa (question 1a), we first used a *Multiple Co-Inertia Analysis* (MCOIA, Bady et al. 2004) to have a visual overview of the correlations between taxonomic groups, and to detect overall compositional (all groups confounded) structures in our data. Abundance and occurrence data were Hellinger-transformed (Legendre & Gallagher 2001) prior to all analyses to avoid inflating the weight of highly abundant taxa in our analyses.

We then performed "classical" *Co-inertia Analysis* (COIA, Chessel & Mercier 1993, Dolédec & Chessel 1994) to quantify pairwise associations of composition between data tables. Conceptually, COIA is very close to the *Procrustes Analysis* and usually leads to redundant results (Digby and Kempton 1987, Dray et al. 2003a, Legendre and Legendre 2012). Both methods measure the co-structure of two data tables among common sampling sites (Dray et al. 2003b). More specifically, in COIA, the co-inertia between two hyper-volumes of *n* dimensions (e.g. *n* eigenvectors from PCA or PCoA) corresponds to the sum of squares of the co-variances between all pairs of variables from both volumes (Dray et al. 2003b).

Unlike *Co-correspondence analysis* (COCA, ter Braak and Schaffers 2004), or constrained ordinations like *Canonical Correspondence Analysis* (CCA, ter Braak 1986) or *Redundancy Analysis* (RDA, Rao 1964), COIA and Procrustes have the advantage of being applicable when

the number of variables far exceeds the number of sampling sites (Legendre and Legendre 2012). However, despite its great potential, COIA remains poorly used and should deserve further consideration in ecological analyses.

Here, we chose COIA instead of Procrustes analysis as the former was used to answer the second question that we addressed in the introduction (see next section). Nevertheless, we also performed Procrustes analyses (calculation of the Procrustes residual sum of square to quantify association between data tables; Jackson 1995) as a complementary approach to further verify consistency in our results.

Prior to performing COIA, abundance and occurrence data were Hellinger-transformed (Legendre and Gallagher 2001) to reduce the weight of overly abundant taxa in each group. As a second option, we also transformed the taxonomic abundance/occurrence data into presence-absence in each group. Although the latter approach removes useful compositional information (MacKenzie 2005), it is worth being explored as presence-absence data are much easier and quicker to collect on the field and many datasets of community composition consist of taxonomic presence-absence (Joseph et al. 2006).

For each pair of species group matrices, we then performed a principal coordinate analysis (PCoA) to obtain vectors representing at least 90% of the inertia of each group. Euclidean distances were used for Hellinger transformed abundance/occurrence data (Legendre and Gallagher 2001), while 1-Jaccard similarity was used for presence absence data (see e.g. Zhang et al. 2016 for Procrustes analyses) in the PCoA.

The co-inertia between the two groups was then measured using the *RV coefficient* (Robert and Escoufier 1976), adjusted following Mayer et al. (2011) to avoid biases due to different numbers of variables between matrices. The RV coefficient varies between 0 and 1, a value

of 1 indicating that both data tables are perfectly correlated. The RV is actually a generalized version of the coefficient of determination in linear regression models (Legendre and Legendre 2012).

To test RV values while taking spatial autocorrelation into account, we compared the observed coefficients with 999 null values obtained using *Moran Spectral Randomizations* (MSR, Wagner & Dray 2015). MSR is a flexible method providing a way to generate artificial variables displaying spatial structures that accurately reproduce structures mimicking those of the original variables. To do so, the method first consists in fitting *Moran Eigenvector's Maps* (MEM, Dray et al. 2006) to the observed data, in order to detect spatial patterns at multiple spatial scales. MEM correspond to eigenvectors obtained by performing a *Principal Coordinates Analysis* on a spatial weighting matrix (see Dray et al. 2006 for details), which model multi-scale spatial structures of univariate or multivariate data tables. Here we used MEM to model spatial structures in the multivariate abundance variation in each of the five species group and in the soil variables' data. More concretely, the MEM method generates *n*-1 eigenvectors (where *n* corresponds to the number of plots) from a doubly-centred spatial weighting matrix generated from the spatial coordinates of the plots (see details in Dray et al. 2006). The spatial weighting matrix (SWM) results from the Hadamard product of a matrix of connectivity (A) and a matrix of weighted spatial distance (B) between plots. Connectivities in A are usually defined according to the spatial configuration of the sampling design, or chosen according to underlying hypotheses regarding the links between plots. Here we tested connectivities defined by *Gabriel's graph* and *minimum spanning tree*, two widely used models that have been proved well-suited for highly discontinuous or, like in our case, nested sampling design consisting of distant clusters (sites) of plots (Bauman et al. 2018a). Note that the distance-based MEM (db-MEM, Dray et al. 2006), which is also widely

used, is better suited for regular sampling designs only, and was therefore not used here (Bauman et al. 2018a). Spatial distances in B remained either untransformed or weighted by a function decreasing linearly with the distance. Thus two sets of n-1 MEM eigenvectors were generated, based on the two types of SWM produced by the Hadamard product of (**1**) **A** = Gabriel's connectivities × **B** = spatial distance (un-transformed) and (**2**) **A** = Gabriel's connectivities × **B** = spatial distance weighted by a function decreasing linearly with the distance. For each of these two sets of MEM, the subset of MEM that best explain each response matrix was obtained by using a forward selection with double stopping criterion (Blanchet et al. 2008), a method that has been shown to be highly powerful and accurate for selecting MEM (Bauman et al. 2018b). We took the warnings of Bauman *et al*. (2018b) into account to avoid type I error inflation when optimising the selection of MEM.

Based on information of the spatial relations (matrices A and B) between plots in the SWM that produced the best subset of MEM, we used the MSR method to generate artificial multivariate data table that reproduced the spatial structures in the original table with good accuracy. More specifically, the MSR first uses a linear combination of MEM variables to model multi-scale spatial structures in the data table. It then uses the detected structures in a conditional randomisation procedure that preserves the original structure in the data. This allows testing a relation with another set of variables (another data table) while maintaining a correct type I error rate (see Wagner and Dray 2015 for details). The method therefore takes spatial autocorrelation into account in our data but it also de-correlates null RV values from soil variables (and thus allows testing whether RV values are significant, independently from soil conditions).

Here, we found that the same MEM eigenvectors obtained on the observed abundance data were selected in each randomised data tables produced by the MSR (not shown), hence demonstrating that the latter method was able to accurately reproduce the original spatial structures in our data tables, and thus further confirming that the method is applicable for a wide variety of sampling configuration, even with a highly clustered plots' distribution like in our study.

To test the RV values calculated for each pair of data table, a *p*-value was computed as the proportion of null RV values from co-inertia analyses (or null residual sum of square values for Procrustes analyses), obtained using 999 MSR-randomised data tables (for one table in the pair), equal to or larger than the observed RV value for a one-tailed test in the upper tail (Hope 1968, Legendre and Legendre 2012). The RV coefficient was considered significant when no more than 5% of the null RV values were equal to or higher than the observed value. A significant RV between two groups would suggest that both groups are good proxies for each other in terms of species composition.

Testing coordinated response to soil variables for each pair of groups

In order to provide answers to questions 2a and 2b, we used the environmental data and the couples of co-inertia axes (see Fig. 1 in Dray et al. 2003a) obtained for each pair of taxonomic groups. These axes are similar to those obtained in the *Co-Correspondence Analysis* (ter Braak and Schaffers 2004). For each pair of groups, we chose the ten first couples of co-inertia axes, which always explained at least 75% of the overall co-inertia between the two groups. As we found that each couple of co-inertia axes were highly correlated to each other (*r*-Pearson always > 0.99), we only kept a single axis in each pair, which therefore led to a total of ten axes per pair of taxonomic groups.

We then computed the R^2 value (adjusted to account for the number of explanatory variables) of a RDA of the co-inertia axes on the set of all soil variables. This value was tested by comparing it with 999 null values obtained using the MSR method described above to account for spatial autocorrelation in the residuals of the RDA model. Tests were considered significant if less than five percent of the null values were higher than the observed one. The relative effect of each individual variable was quantified using the sum of weighted AIC_c (hereafter, SWA) from *Model Averaging with Multivariate GLM* (Nakamura et al. 2015). The R^2 test and SWA values obtained for each pair of taxonomic group allowed testing whether the co-inertia between two groups was explained by soil heterogeneity, and if so, which soil variable(s) best explained coordinated response between the groups.

MEM were used to test at which spatial scale(s) the overall soil heterogeneity and each individual soil variable influenced the co-inertia among pairs of groups (MEM eigenvalues are directly correlated to Moran's *I* (Moran 1953) and therefore to the spatial scales at which variables are structured). The highest eigenvalues reflect broad-scale spatial autocorrelation while MEM with relatively lower eigenvalues model smaller-scale structures. We thus selected with a forward selection (Blanchet et al. 2008) the set of MEM variables that significantly described spatial structures in the overall soil variables' table and each variable taken individually. Based on visual representation of scores from each significant MEM variable, we assigned the following nominal type of spatial structure for each variable: "Broad" (B) if all selected MEM vectors mostly represent differences of scores among sites, "Fine" (F) if the vectors mostly represent differences of scores among plots within sites, both "Broad" and "Fine" (BF) if they represent differences of scores among and within sites, and "ns" if no structure was detected. It is worth noting that the absence of significant structure did not necessarily mean an absence of spatial dependence among plots, but that the limits

of spatial resolution of our sampling design did not allow modelling these dependences using MEM.

All analyses were performed in the R statistical environment (R Development Core Team 2018), using packages *car* (Fox & Weisberg 2011), *ade4* (Dray & Dufour 2007), *adespatial* (Dray et al. 2018), *usdm* (Naimi et al. 2014), *cocorresp* (Simpson 2009), *MatrixCorrelation* (Indahl et al. 2017), *vegan* (Oksanen et al. 2018), *ape* (Paradis et al. 2004) and *mglmn* (Katabuchi & Nakamura 2015).

References for Appendix S2

Bady, P., Dolédec, S., Dumont, B., Fruget, J.-F. (2004). Multiple co-inertia analysis: a tool for assessing synchrony in the temporal variability of aquatic communities. Cr. Biol., 327(1), 29–36.

Bauman, D., Drouet, T., Fortin, M.J. and Dray, S. (2018a). Optimizing the choice of a spatial weighting matrix in eigenvector-based methods. Ecology, in press.

Bauman, D., Drouet T., Dray, S. & Vleminckx, J. (2018b). Disentangling good from bad practices in the selection of spatial or phylogenetic eigenvectors. Ecography, 41, 1–12, 2018.

Blanchet, F.G., Legendre, P. & Borcard, D. (2008). Forward selection of explanatory variables. Ecology, 89, 2623–2632.

Chessel, D. & Mercier, P. (1993). Couplage de triplets statistiques et liaisons espèces—environnement. Pages 15–43 in J. D. Lebreton and B. Asselain, editors. Biométrie et Environnement. Masson, Paris, France.

Digby, P.G.N. & Kempton, R.A. (1987). Multivariate analysis of ecological communities. Chapman and Hall, London, UK.

Dolédec, S. & Chessel, D. (1994). Co-inertia analysis: an alternative method for studying species–environment relationships. Freshwater Biol., 31, 277–294.

Dray, S., Chessel, D. & Thioulouse, J. (2003a). Procrustean co-inertia analysis for the linking of multivariate data sets. Ecoscience, 10, 110–119.

Dray, S. Chessel, D. & Thioulouse, J. (2003b). Co-inertia analysis and the linking of ecological tables. Ecology, 84(11), 3078–3089.

Dray, S., Legendre, P. & Peres-Neto, P.R. (2006). Spatial modelling: a comprehensive framework for principal coordinate analysis of neighbour matrices (PCNM). Ecol. Model., 196, 483–493.

Dray, S. and Dufour, A.B. (2007): The ade4 package: implementing the duality diagram for ecologists. J. Stat. Softw. 22(4), 1–20.

Dray, S., Bauman, D., Blanchet, G., Borcard, D., Clappe, S., Guenard, G. et al. (2018). adespatial: Multivariate Multiscale Spatial Analysis. R package version 0.2-0. https://CRAN.R-project.org/package=adespatial.

Fox, J. & Weisberg, S. (2011). car: An R Companion to Applied Regression, Second Edition. Thousand Oaks CA: Sage. URL: http://socserv.socsci.mcmaster.ca/jfox/Books/Companion.

Hope, A.C.A. 1968. A simplified Monte Carlo significance test procedure. J. Roy. Statist. Soc. Ser. B, 30, 582–598.

Indahl, U.G., Naes, T. & Liland, K.H. (2017). A similarity index for comparing coupled matrices. J. Chemometr. 32(10), e3049.

Jackson, D.A. (1995). PROTEST: a PROcrustean Randomization TEST of community environment concordance. Ecoscience, 2, 297–303.

Joseph, L.N., Field, S.A., Wilcox, C. & Possingham H.P. (2006). Presence-absence versus abundance data for monitoring threatened species. Conserv. Biol., 20(6), 1679–1687.

Katabuchi M. & Nakamura A. (2015). mglmn: Model Averaging for Multivariate GLM with Null Models. R package version 0.0.2. https://CRAN.R-project.org/package=mglmn.

MacKenzie, D.I. (2005). What are the issues with presence-absence data for wildlife managers. J. Wildlife Manag., 69(3), 849–860.

Mayer, C.D., Lorent, J. & Horgan, G.W. (2011). Exploratory analysis of multiple omics datasets using the adjusted RV coefficient. Stat. Appl. Genet. Mol. Biol., 10, article 14.

Moran, P.A.P. (1953). The statistical analysis of the Canadian lynx cycle. II. Synchronization and meteorology. Aust. J. Zool., 1, 291–298.

Naimi B., Hamm N., Groen T.A., Skidmore A.K. AND Toxopeus A.G. (2014). "Where is positional uncertainty a problem for species distribution modelling." Ecography, 37, 191–203. doi: 10.1111/j.1600-0587.2013.00205.x (URL: http://doi.org/10.1111/j.1600-0587.2013.00205.x).

Legendre, P. & Gallagher, E.D. (2001). Ecologically meaningful transformations for ordination of species data. Oecologia 129: 271–280.

Legendre, P. & Legendre, L. (2012). Numerical Ecology, 3rd English edn. Elsevier Science BV, Amsterdam.

Oksanen, J., Blanchet, F.G., Friendly, M., Kindt, R., Legendre, P., McGlinn, D. et al. (2018). vegan: Community Ecology Package. R package version 2.5-2. https://CRAN.R-project.org/package=vegan.

Paradis E., Claude J. & Strimmer K. 2004. APE: analyses of phylogenetics and evolution in R language. Bioinformatics 20, 289–290.

Peres-Neto, P.R., Legendre, P., Dray, S. & Borcard, D. (2006). Variation partitioning of species data matrices: estimation and comparison of fractions. Ecology, 87, 2614–2625.

Rao, C. R. (1964). The use and interpretation of principal component analysis in applied research. Sankhya A, 26, 329–359.

Robert, P. & Escoufier, Y. (1976). "A Unifying tool for linear multivariate statistical methods: the RV-coefficient", Appl. Stat., 25(3), 257–265.

Simpson, G.L. (2009). cocorresp: Co-correspondence analysis ordination methods. (R package version 0.3-0). (http://cran.r-project.org/package=analogue).

ter Braak, C.J.F. (1986). Canonical correspondence analysis: a new eigenvector technique for multivariate direct gradient analysis. Ecology, 67, 1167–1179.

ter Braak, C.J.F., & Schaffers, A.P. (2004). Co-correspondence analysis: a new ordination method to relate two com-munity compositions. Ecology, 85, 834–846.

Van Buuren S. & Groothuis-Oudshoorn K. (2011). mice: Multivariate Imputation by Chained Equations in R. Journal of Statistical Software, 45(3), 1–67. URL https://www.jstatsoft.org/v45/i03/.

Wagner, H. H. & Dray, S. (2015). Generating spatially constrained null models for irregularly spaced data using Moran spectral randomization methods. Methods Ecol. Evol., 6, 1169–1178.

Zhang, K., Lin, S., Ji, Y., Yang, C., Wang, X., Yang, C. et al. (2016). Plant diversity accurately predicts insect diversity in two tropical landscapes. Mol. Ecol., 25, 4407–4419. doi:10.1111/mec.13770

Figures S1 to S7 of the article "*Coordinated community structure across five taxonomic groups in Amazonian rainforests*" – Vleminckx et al.

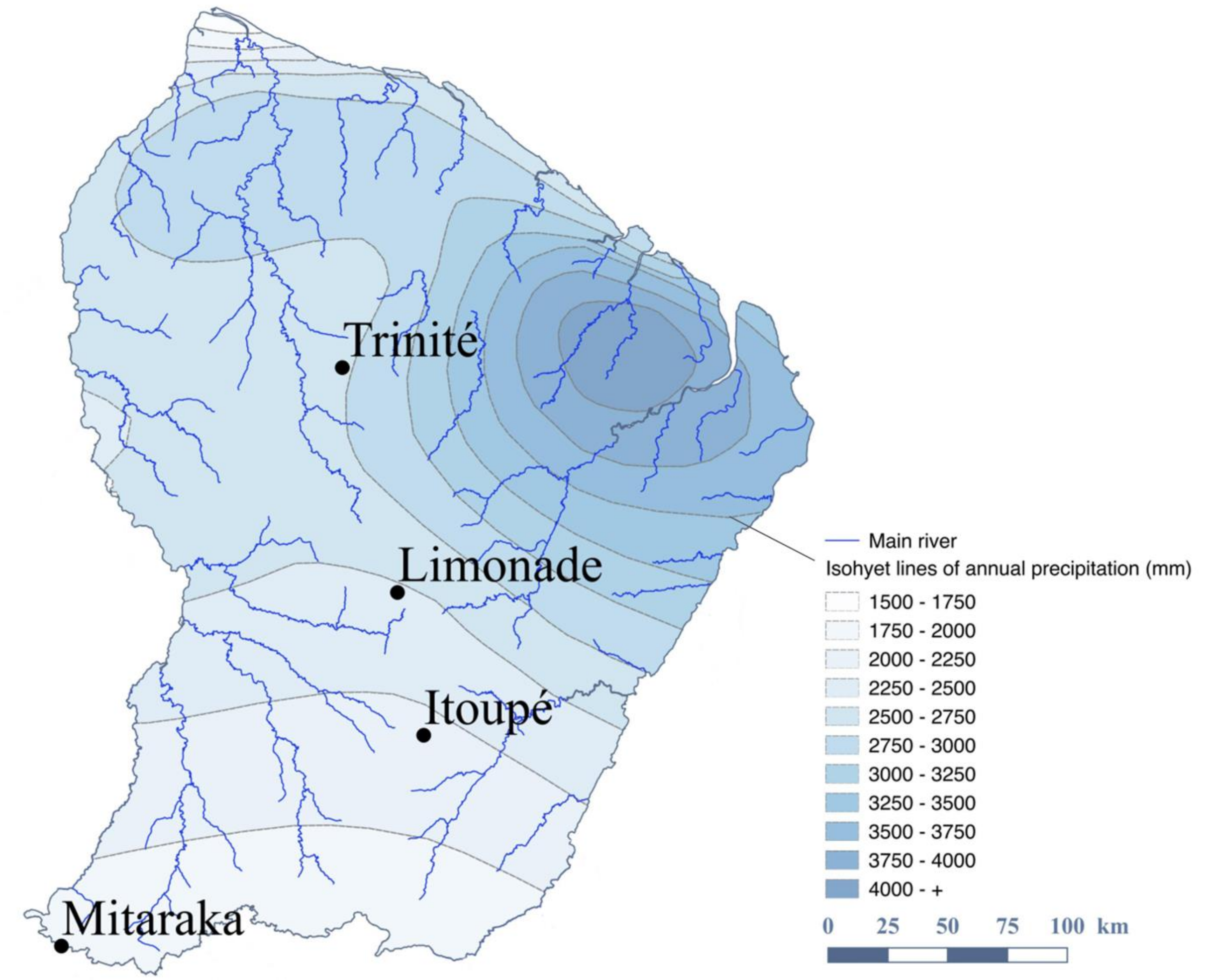

Fig. S1. Geographic map of French Guiana, showing the location of the four inventory sites (*Trinité*, *Limonade*, *Itoupé* and *Mitaraka*).

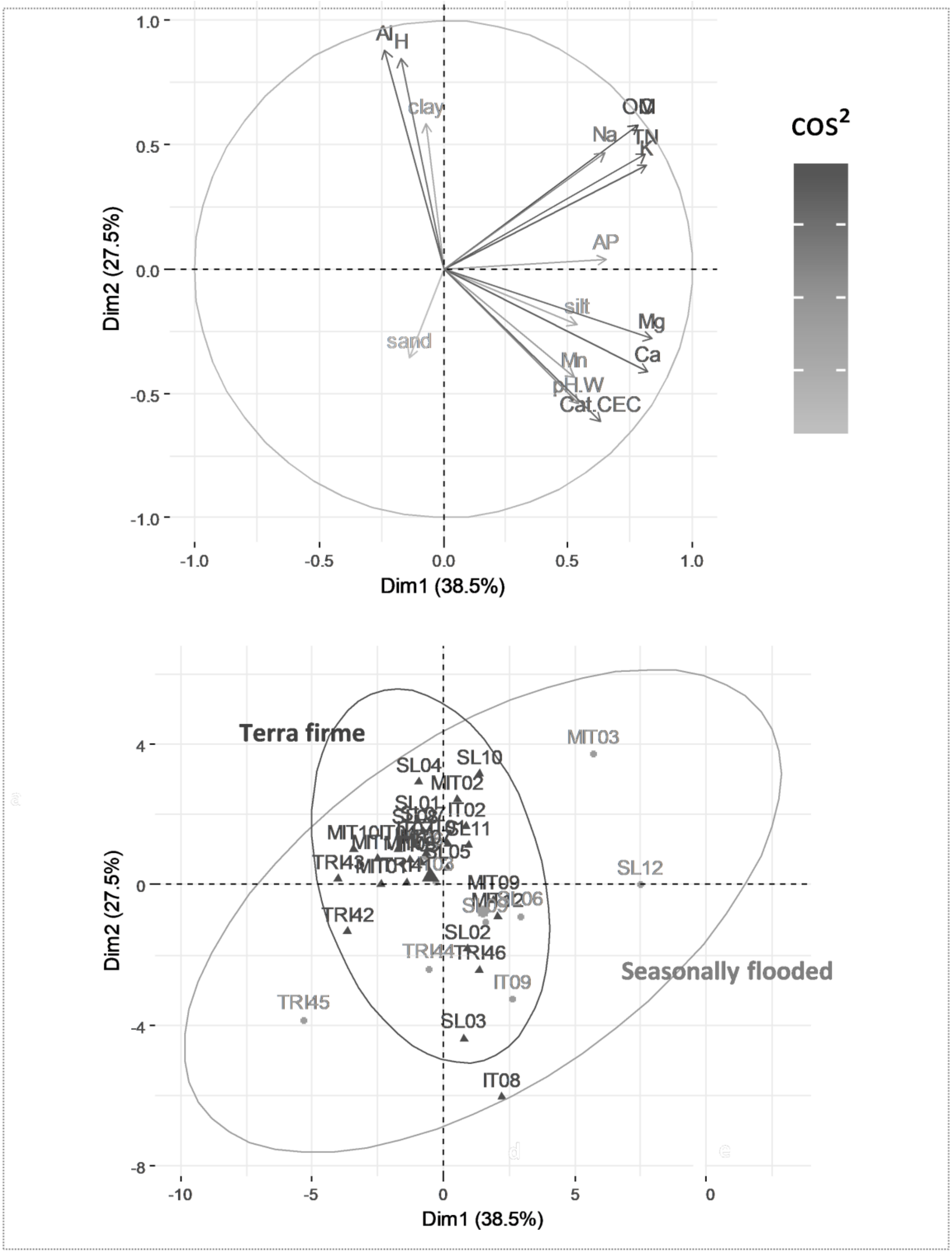

Fig. S2. Top: correlations between soil variables on axes 1-2 of a principal component analysis performed using data from all plots (n = 36), including those on seasonally flooded forest. Bottom: projection of the plots on axes 1-2 of the same analysis. The most excentric plots – TRI45, MIT03, SL12 and IT08 – correspond to plots located on seasonally flooded forest.

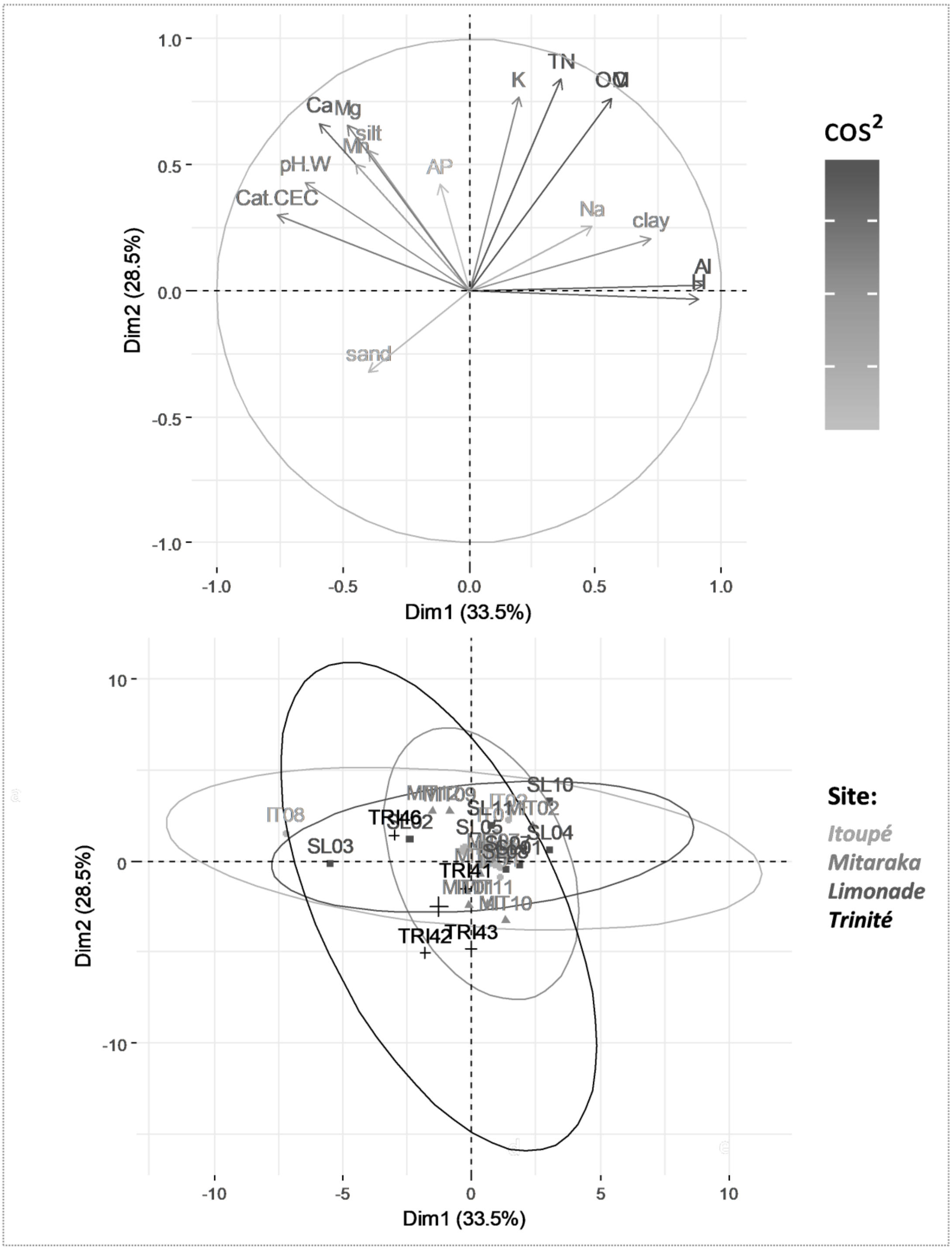

Fig. S3. Top: correlations between soil variables on axes 1-2 of a principal component analysis performed after removing the nine plots located on seasonally flooded forest (see Table 2). Bottom: projection of the plots on axes 1-2 of the same analysis.

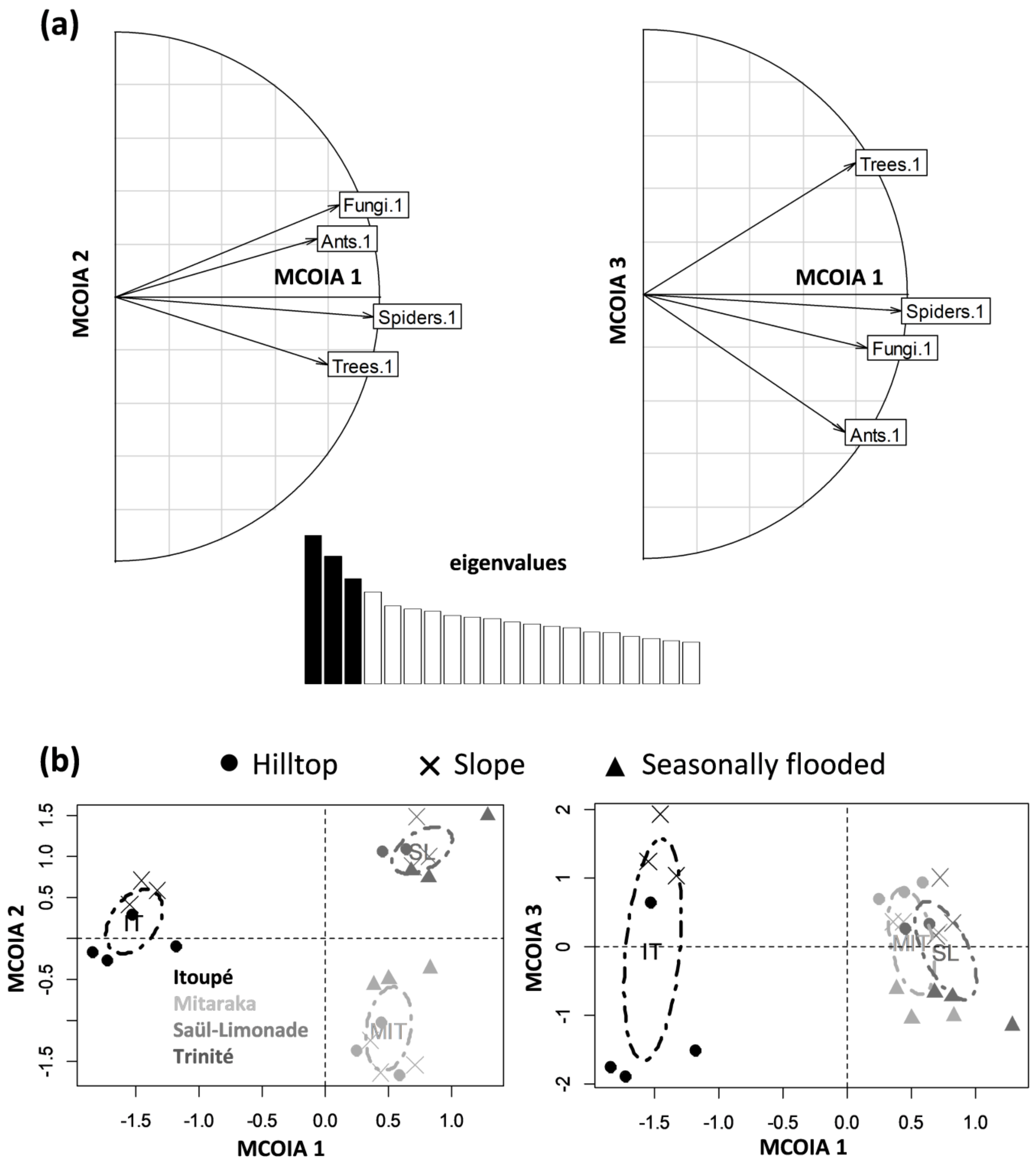

Figure S4. (**a**) Correlations between the first axes of separate PCA (performed on **four species groups** – **trees, ants, spiders and fungi** – present in **three sites**) and the three first axes of the MCOIA (axes 1-2 and 1-3). (**b**) Projection of plot scores on axes 1-2 and axes 1-3 of the MCOIA, emphasizing the three sites where compositional data was available for the four groups (Mitaraka = MIT, Saül-Limonade = SL and Itoupé = IT) as well as topographical habitats (hilltop, slope and seasonally flooded). Histograms represent the eigenvalues of the MCOIA axes.

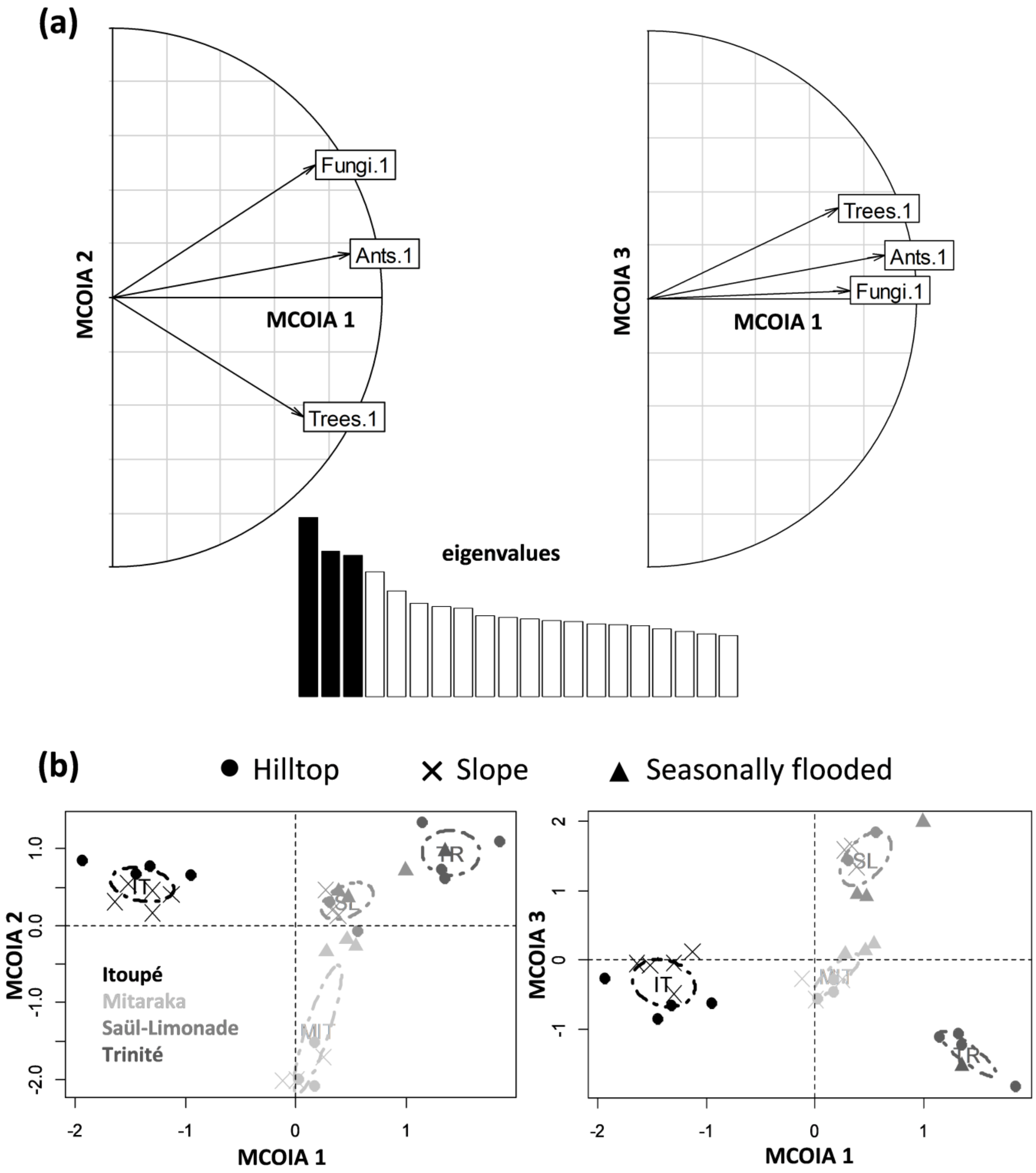

Figure S5. (**a**) Correlations between the first axes of separate PCA (performed on **three species groups – trees, ants, fungi** – present in **four sites**) and the three first axes of the MCOIA (axes 1-2 and 1-3). (**b**) Projection of plot scores on axes 1-2 and axes 1-3 of the MCOIA, emphasizing the four sites where compositional data was available for the four groups (Mitaraka = MIT, Saül-Limonade = SL, Itoupé = IT and Trinité = TR) as well as topographical habitats (hilltop, slope and seasonally flooded). Histograms represent the eigenvalues of the MCOIA axes.

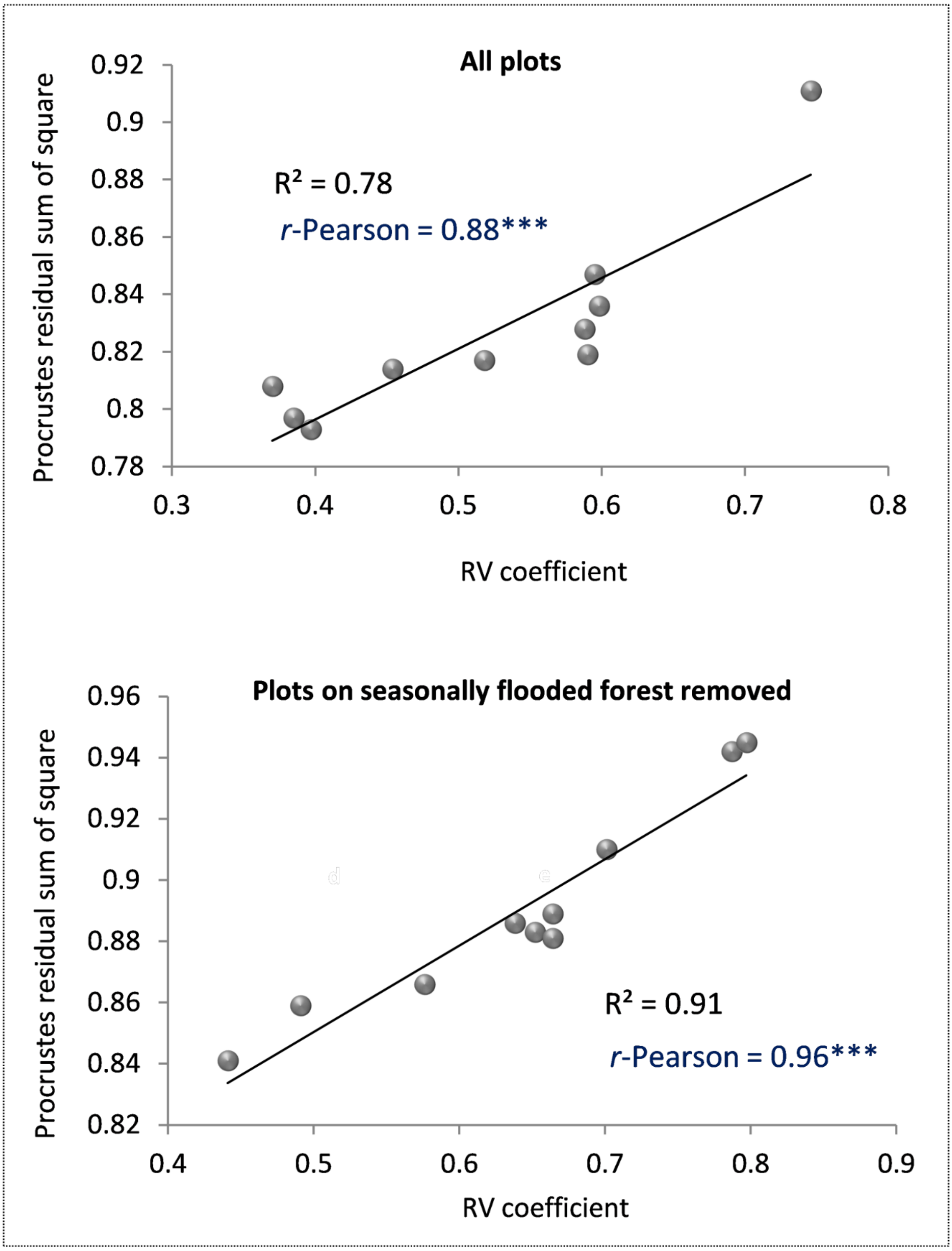

Fig. S6. Scatterplot showing the correlation between RV values of the co-inertia analysis and the residual sum of square values of the Procrustes analysis (data untransformed) obtained **using abundance/species occurrence data**, and without including values calculated with soil data tables. The *r*-Pearson correlation value was tested using a t-test implemented in the "*cor.test*" R function (package "*stats*").

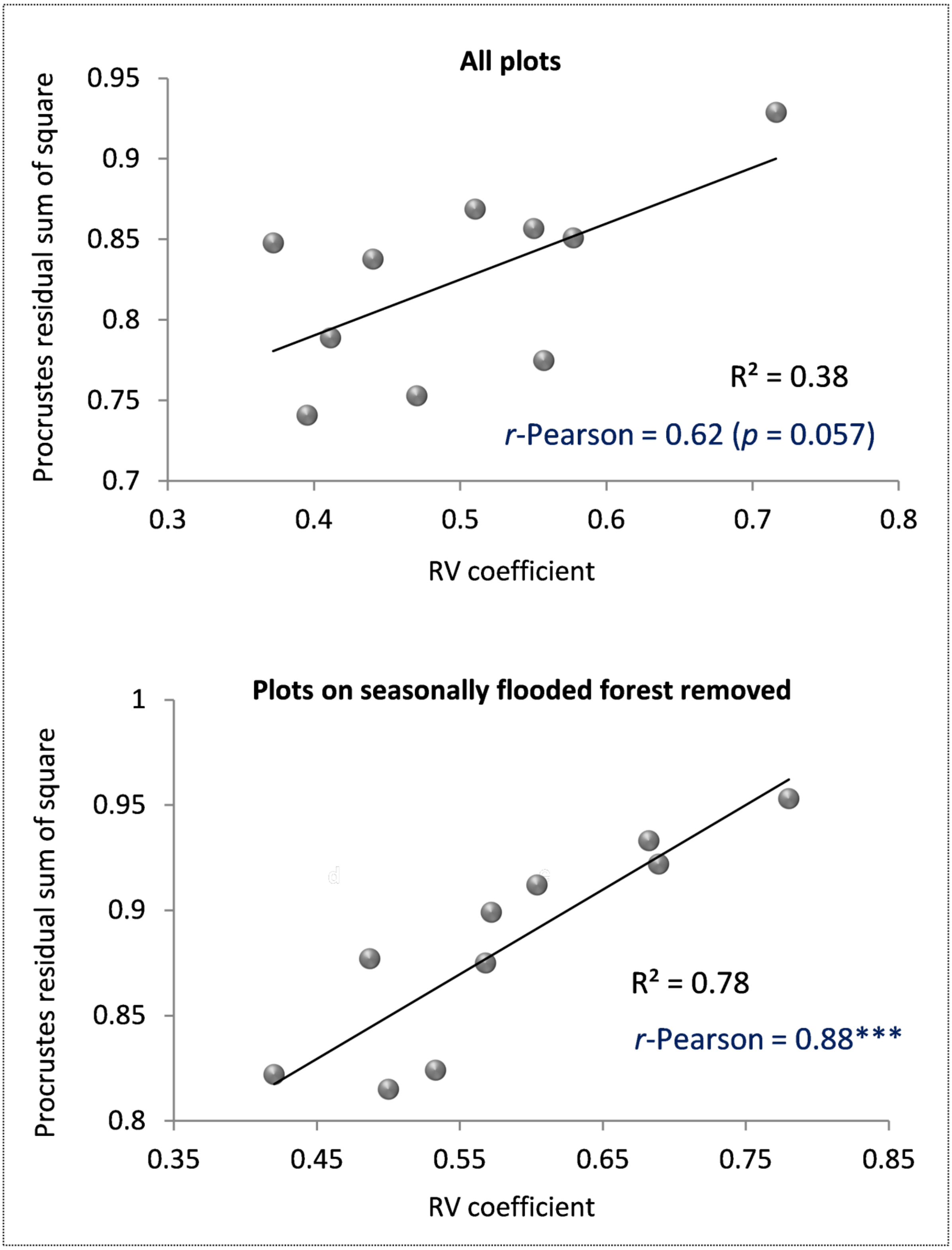

Fig. S7. Scatterplot showing the correlation between RV values of the co-inertia analysis and the residual sum of square values of the Procrustes analysis (data untransformed) obtained **using presence-absence data**, and without including values calculated with soil data tables. The *r*-Pearson correlation value was tested using a t-test implemented in the "*cor.test*" R function (package "*stats*").

Tables S1 to S4 of the article "*Coordinated community structure across five taxonomic groups in Amazonian rainforests*" – Vleminckx et al.

Table S1. ***Adjusted RV values of co-inertia analyses***, calculated for each pair of taxonomic groups (shaded in grey), and between each taxonomic group and soil variables, obtained using ***abundance/species occurrence data***. Values were calculated using all plots or without plots located on seasonally flooded forest (SF). Asterisks indicate whether values were significant (in bold) according to the MSR test: **P* < 0.05; ***P* < 0.01; ****P* < 0.001. The last line represents the mean (± standard deviation) of RV values per group.

	Soil	Trees	Ants	Spiders	Fungi	mean
All plots						
Trees	**0.623*****					0.470 (± 0.149)
Ants	**0.601*****	**0.746*****				0.468 (± 0.129)
Spiders	**0.411*****	**0.598*****	**0.518*****			0.566 (± 0.035)
Fungi	**0.360*****	**0.454*****	**0.385****	**0.590***		0.478 (± 0.128)
Earthworms	**0.378*****	**0.397***	**0.370****	**0.595****	**0.588****	0.466 (± 0.147)
SF plots removed						
Trees	**0.688*****					0.533 (± 0.114)
Ants	**0.656****	**0.787*****				0.564 (± 0.147)
Spiders	**0.733****	**0.652*****	**0.664*****			0.617 (± 0.031)
Fungi	**0.749*****	**0.491****	0.441	0.576		0.496 (± 0.131)
Earthworms	**0.541***	**0.664***	**0.701***	0.638	**0.797****	0.596 (± 0.077)

Table S2. ***Procrustes residual sum of square values*** calculated for each pair of taxonomic groups (shaded in grey), and between each taxonomic group and soil variables, obtained ***using abundance/species occurrence data***. Values were calculated using all plots or without plots located on seasonally flooded forest (SF). Asterisks indicate whether values were significant (in bold) according to the MSR test: **P* < 0.05; ***P* < 0.01; ****P* < 0.001. The last line represents the mean (± standard deviation) of RV values per group.

	Soil	Trees	Ants	Spiders	Fungi	mean
All plots						
Trees	**0.496*****					0.805 (± 0.498)
Ants	**0.514*****	**0.911*****				0.814 (± 0.042)
Spiders	**0.410*****	**0.836*****	**0.817*****			0.825 (± 0.014)
Fungi	**0.351****	**0.814****	**0.797****	**0.819***		0.799 (± 0.030)
Earthworms	**0.345***	**0.793***	**0.808****	**0.847*****	**0.828****	0.808 (± 0.042)
SF plots removed						
Trees	**0.578*****					0.856 (± 0.034)
Ants	**0.587*****	**0.942*****				0.864 (± 0.049)
Spiders	**0.652*****	**0.883*****	**0.881*****			0.864 (± 0.016)
Fungi	**0.664*****	**0.859*****	**0.841***	**0.866****		0.833 (± 0.034)
Earthworms	0.474	**0.889****	**0.910****	**0.886****	**0.945*****	0.870 (± 0.024)

Table S3. ***Adjusted RV values of co-inertia analyses***, calculated for each pair of taxonomic groups (shaded in grey), and between each taxonomic group and soil variables, obtained using ***presence-absence data***. Values were calculated using all plots or without plots located on seasonally flooded forest (SF). Asterisks indicate whether values were significant (in bold) according to the MSR test: *$P < 0.05$; **$P < 0.01$; ***$P < 0.001$. The last line represents the mean (± standard deviation) of RV values per group.

	Soil	Trees	Ants	Spiders	Fungi	mean
All plots						
Trees	**0.576*****					0.554 (± 0.066)
Ants	**0.599*****	**0.716*****				0.473 (± 0.124)
Spiders	**0.323*****	**0.557*****	**0.470****			0.456 (± 0.070)
Fungi	**0.396*****	**0.550*****	**0.440*****	0.395		0.476 (± 0.091)
Earthworms	**0.336*****	**0.510***	0.372	0.411	**0.577***	0.463 (± 0.091)
SF plots removed						
Trees	**0.662*****					0.567 (± 0.081)
Ants	**0.639****	**0.780*****				0.514 (± 0.128)
Spiders	0.633	0.533	0.500			0.452 (± 0.068)
Fungi	**0.754*****	**0.572****	**0.487***	0.420		0.471 (± 0.121)
Earthworms	0.504	0.604	0.682	0.568	0.689	0.528 (± 0.062)

Table S4. ***Procrustes residual sum of square values***, calculated for each pair of taxonomic groups (shaded in grey), and between each taxonomic group and soil variables, obtained using ***presence-absence data***. Values were calculated using all plots or without plots located on seasonally flooded forest (SF). Asterisks indicate whether values were significant (in bold) according to the MSR test: **P* < 0.05; ***P* < 0.01; ****P* < 0.001. The last line represents the mean (± standard deviation) of RV values per group.

	Soil	Trees	Ants	Spiders	Fungi	mean
All plots						
Trees	**0.433*** **					0.857 (± 0.063)
Ants	**0.490*** **	**0.929*** **				0.842 (± 0.072)
Spiders	**0.356** **	**0.775** **	**0.753** **			0.764 (± 0.022)
Fungi	**0.365** **	**0.857*** **	**0.838*** **	**0.741***		0.822 (± 0.054)
Earthworms	**0.342***	**0.869*** **	**0.848** **	0.789	**0.851*** **	0.839 (± 0.035)
SF plots removed						
Trees	**0.541*** **					0.897 (± 0.054)
Ants	**0.576*** **	**0.953*** **				0.894 (± 0.062)
Spiders	**0.597***	0.824	**0.815***			0.834 (± 0.028)
Fungi	**0.650** **	**0.899*** **	**0.877** **	0.822		0.880 (± 0.043)
Earthworms	0.464	**0.912*** **	**0.933*** **	**0.875** **	**0.922***	0.911 (± 0.025)
